# Changing nutrient cycling in Lake Baikal, the world’s oldest lake

**DOI:** 10.1073/pnas.2013181117

**Published:** 2020-10-19

**Authors:** George E. A. Swann, Virginia N. Panizzo, Sebastiano Piccolroaz, Vanessa Pashley, Matthew S. A. Horstwood, Sarah Roberts, Elena Vologina, Natalia Piotrowska, Michael Sturm, Andre Zhdanov, Nikolay Granin, Charlotte Norman, Suzanne McGowan, Anson W. Mackay

**Affiliations:** ^a^School of Geography, University of Nottingham, Nottingham NG7 2RD, United Kingdom;; ^b^Physics of Aquatic Systems Laboratory, School of Architecture, Civil and Environmental Engineering, Ecole Polytechnique Fédérale de Lausanne, CH-1015 Lausanne, Switzerland;; ^c^Geochronology and Tracers Facility, British Geological Survey, Nottingham NG12 5GG, United Kingdom;; ^d^Canada Centre for Inland Waters, Environment and Climate Change Canada, Burlington, ON L7S 1A1, Canada;; ^e^Institute of Earth’s Crust, Siberian Branch, Russian Academy of Sciences, 664033 Irkutsk, Russia;; ^f^Division of Geochronology and Environmental Isotopes, Institute of Physics–Centre for Science and Education, Silesian University of Technology, 44-100 Gliwice, Poland;; ^g^Eidgenössische Anstalt für Wasserversorgung, Abwasserreinigung und Gewässerschutz–Eidgenössische Technische Hochschule, Swiss Federal Institute of Aquatic Science and Technology, 8600 Dübendorf, Switzerland;; ^h^Limnology Institute, Siberian Branch, Russian Academy of Sciences, 6644033 Irkutsk, Russia;; ^i^Environmental Change Research Centre, Department of Geography, University College London, London WC1E 6BT, United Kingdom

**Keywords:** Siberia, limnology, climate, ecosystem, endemic

## Abstract

Lake Baikal, lying in a rift zone in southeastern Siberia, is the world's oldest, deepest, and most voluminous lake that began to form over 30 million years ago. Cited as the “most outstanding example of a freshwater ecosystem” and designated a World Heritage Site in 1996 due to its high level of endemicity, the lake and its ecosystem have become increasingly threatened by both climate change and anthropogenic disturbance. Here, we present a record of nutrient cycling in the lake, derived from the silicon isotope composition of diatoms, which dominate aquatic primary productivity. Using historical records from the region, we assess the extent to which natural and anthropogenic factors have altered biogeochemical cycling in the lake over the last 2,000 y. We show that rates of nutrient supply from deep waters to the photic zone have dramatically increased since the mid-19th century in response to changing wind dynamics, reduced ice cover, and their associated impact on limnological processes in the lake. With stressors linked to untreated sewage and catchment development also now impacting the near-shore region of Lake Baikal, the resilience of the lake’s highly endemic ecosystem to ongoing and future disturbance is increasingly uncertain.

## Significance

Lake Baikal (Siberia) is the world’s oldest and deepest lake and a UNESCO World Heritage Site. Containing an exceptionally high level of biodiversity and endemism, in addition to a fifth of global freshwater not stored in ice sheets, the lake has been cited by UNESCO as the “most outstanding example of a freshwater ecosystem.” Using geochemical and climate data, we demonstrate that rates of nutrient supply to the lake’s photic zone have risen to unprecedented levels in the last 2,000 y through the 20th and 21st centuries. Linked to increases in wind speed enhancing deep ventilation, we show that these changes are capable of altering lake primary production and community dynamics, including the balance between endemic and cosmopolitan species.

**Fig. 1.**
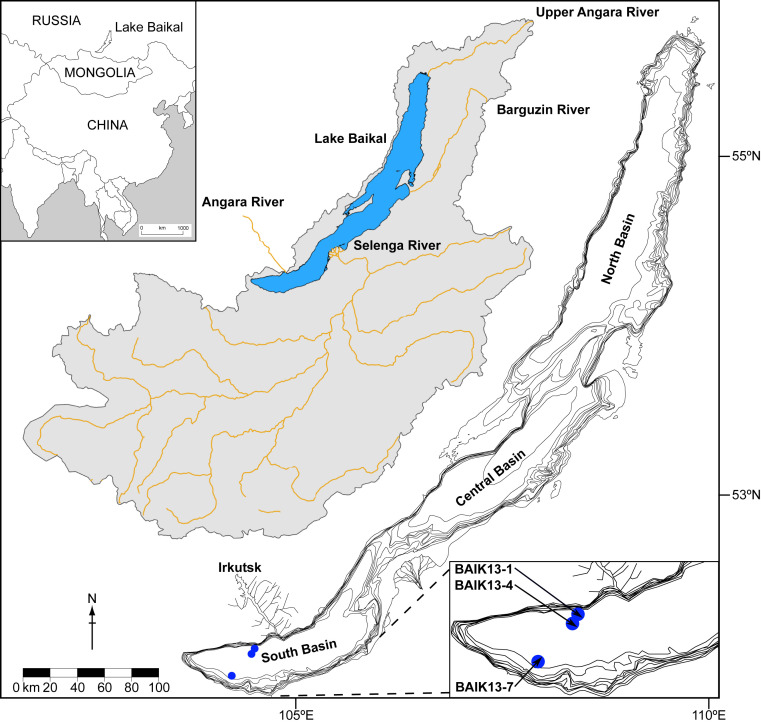
Location of Lake Baikal and its catchment (gray) together with the location of World Meteorological Organization station in Irkutsk, major catchment rivers (brown), coring sites (BAIK13-1, BAIK13-4), and sites providing additional data used in this study (BAIK13-7).

Ancient lakes have long been associated with both high levels of biodiversity and endemicity. However, they are also being threatened by anthropogenic forcings that have led to impacts ranging from the warming of lake waters (1), hydrological modifications (2), increases in aquatic toxicity (3), and declining endemic populations due to introductions of nonnative species (4). With global populations increasingly reliant on large and ancient lakes for ecosystem services, the biodiversity (5) and value of aquatic systems to society (6), particularly in ancient lake systems (7), are at risk. Lake Baikal (Russia) is an exceptional example of an ancient lake (Fig. 1). In addition to containing ∼20% of global surface freshwater, the lake is characterized by its high degree of biodiversity with over 2,500 flora and fauna, the majority of which are endemic (8). This has been attributed to the lake’s age and fully oxygenated water column, driven by seasonal overturning and deep water renewal (9, 10) that sustains an almost completely endemic deep water fauna (8).

Concerns exist over the future health of this unique ecosystem, amid evidence of extensive shoreline eutrophication (11, 12) and climate-induced shifts in primary productivity (13, 14). Together, these changes have impacted organisms ranging from sponges and gastropods to ciliates, flagellates, and algal communities (15). Given the likelihood of future anthropogenic disturbance on Lake Baikal, further disrupting productivity exchanges through the lake’s food web, there is a need to place these contemporary observations into their historical setting. In Lake Baikal, we have evidence that algal communities have undergone rapid multidecadal to multicentennial timescale changes over the last 2,000 y (16). However, there is a need to also gain a clearer insight into how biogeochemical and nutrient cycling has altered over the same timescale, both to contextualize natural and anthropogenic drivers of change and to understand the susceptibility of the lake’s ecosystem to further alteration under different climate states (17). Annual primary productivity in Lake Baikal is ultimately regulated by photic zone nutrient availability, in addition to ice/snow cover, which regulates light availability for photosynthesis (10, 18). Here, by analyzing the silicon isotope composition of diatom silica (δ^30^Si_diatom_), we show that nutrient supply to the surface waters of Lake Baikal has rapidly increased through the 20th and 21st centuries coincident with increased wind-driven Ekman transport and reduced ice cover. These changes in photic zone nutrient availability have the potential to alter resource competition and prey–predator interactions across the lake (15, 19).

## Results and Discussion

**Fig. 2.**
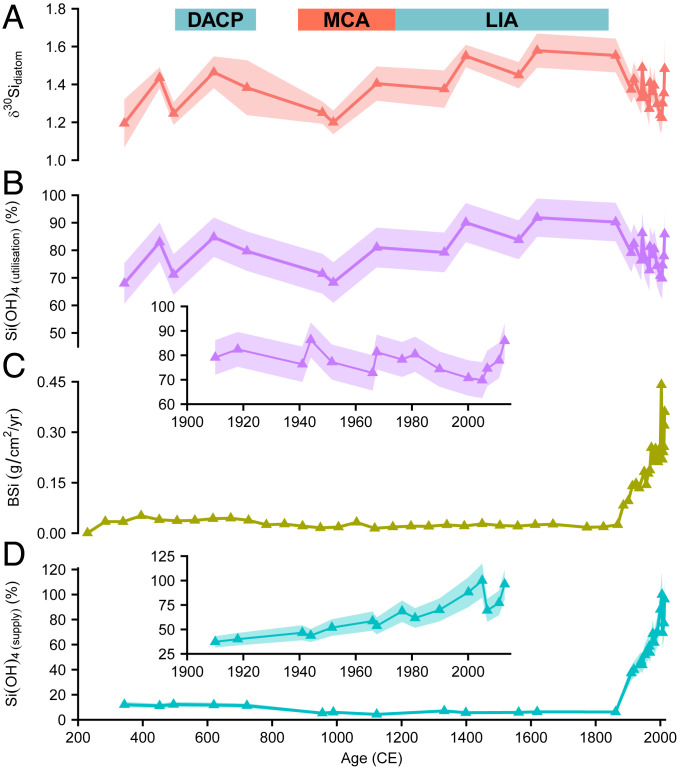
Proxy records from Lake Baikal reflecting changes in silicon cycling in the lake. (*A*) δ^30^Si_diatom_ from Lake Baikal. (*B*) Relative rates of photic zone silicic acid [SiOH_4_] utilization. (*C*) Biogenic silica (BSi) mass accumulation rates (MARs) at core site BAIK13-1 (Fig. 1). (*D*) Changes in photic zone silicic acid supply relative to a value of 100% at 2005 CE. Shaded region for δ^30^Si_diatom_ reflects the absolute analytical uncertainty (2σ) of the isotope analysis. Shaded polygons for silicic acid supply/utilization and BSi MAR reflect the 1σ uncertainty derived from Monte Carlo simulations (10,000 replicates). Age boundaries for the Little Ice Age (LIA) and Medieval Climate Anomaly (MCA) are based on diatom assemblage records of environmental change from Lake Baikal (22). Also shown are age boundaries for the Dark Ages Cold Period (DACP). Subplots in *B* and *D* document changes since 1900 CE.

Our composite δ^30^Si_diatom_ record, from the south basin of Lake Baikal (Figs. 1 and 2*A*), is controlled by changes in the rates at which nutrients (silicic acid [SiOH_4_]) are supplied to the photic zone and the rates at which the same nutrients are utilized by diatoms (unicellular siliceous algae, which dominate primary productivity in Lake Baikal). An increase (decrease) in δ^30^Si_diatom_ could therefore be driven by the following: 1) an increase (decrease) in biogenic silicic acid utilization due to the isotope fractionation associated with this process; 2) a decrease (increase) in nutrient supply to the photic zone, which replenishes the pool of nutrients and their isotope composition; or 3) a combination of these two processes, with their relative magnitudes determining the direction of change in δ^30^Si_diatom_ (e.g., increased rates of both nutrient utilization and supply). These two parameters can be constrained from δ^30^Si_diatom_ using modern Lake Baikal values that account for the δ^30^Si_diatom_ fractionation factor (20), the δ^30^Si composition of deep lake water that dominate intraannual and interannual nutrient supply to the photic zone (δ^30^Si_lake_) (21), and biogenic silica (BSi) mass accumulation rates (MARs) to account for siliceous algal productivity.

For the past 2,000 y, changes in δ^30^Si_diatom_ follow rates of silicic acid utilization, which predominantly varies from 70 to 90% (x̄ = 79%, 1σ = 6.7%). Rates in the 20th and 21st centuries (x̄ = 78%, 1σ = 5.0%) are lower than during the Little Ice Age (LIA) from 1180 to 1840 CE (x̄ = 86%, 1σ = 5.8%) (*P* < 0.05) and Dark Ages Cold Period (DACP) from 500 to 750 CE (x̄ = 82%, 1σ = 3.6%), but similar to the Medieval Climate Anomaly (MCA) from 880 to 1180 CE (x̄ = 74%, 1σ = 6.6%) (Fig. 2*B*). Evidence of a 20th- and 21st-century decline in silicic acid utilization, relative to the LIA, contrasts with evidence of increasing BSi MAR over the same interval seen in sediment cores from across Lake Baikal (16). By constraining utilization as a ratio against BSi MAR from the same core samples, the 20th- and 21st-century decline in silicic acid utilization can be attributed to a progressive increase in the rates at which nutrients (silicic acid) are supplied to the photic zone, with a significant escalation after 1900 CE (*P* < 0.001) (Fig. 2 *C* and *D*). In other words, while absolute rates of siliceous productivity and biomineralization increased (Fig. 2*C*), the rate of nutrient supply to the photic zone occurred at a faster rate than the same nutrients could be biomineralized, leading to the 20th- and 21st-century decrease in relative rates of nutrient utilization.

### Nutrient Cycling in Lake Baikal.

Due to the dissolution of diatoms and other organisms during sinking and the associated remineralization of nutrients into the water column, deep water nitrate, phosphate, and silicate nutrient concentrations are higher than the overlying waters in the epilimnion (9, 23). Intraannual and interannual geochemical cycling in Lake Baikal is therefore primarily regulated by the vertical mixing of nutrient-rich deep waters into the photic zone (23), sustaining high levels of primary productivity within the lake. Due to thermal stratification, seiches and seasonal convective mixing in Lake Baikal do not extend below 300 m (10), while cyclonic induced upwelling is constrained to the upper 400 m of the water column (24). Instead, whole water column vertical mixing is primarily controlled by coastal downwelling, triggered by thermobaric instability in a process known as deep ventilation (9, 10, 25) and balanced by the upwelling of deep water rich in silicon, nitrogen, and phosphate to the photic zone (630 mmol SiO_2_⋅m^−2^⋅y^−1^; 93 mmol NO_3_^−^⋅m^−2^⋅y^−1^; 5 mmol P⋅m^−2^⋅y^−1^) (23).

**Fig. 3.**
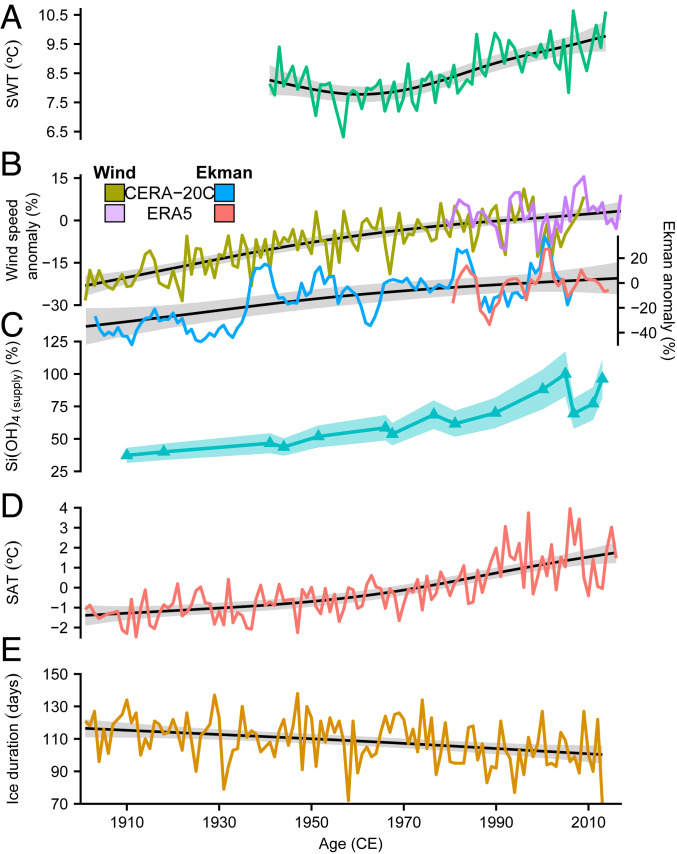
Drivers of silicic acid supply in Lake Baikal. (*A*) Mean surface water temperatures (SWTs) (May to October) from shoreline locations across Lake Baikal (29). (*B*) CERA-20C (32) and ERA5 (33) wind speed reanalysis data from the barycentre of Lake Baikal and modeled 5-y running mean Ekman transport in Lake Baikal during the typical periods of downwelling (May to June and December to January). Both datasets are shown as anomalies relative to a baseline period of 1990 to 2000 CE. (*C*) Changes in photic zone silicic acid supply relative to a value of 100% at 2005 CE with the shaded polygon reflect the 1σ uncertainty derived from Monte Carlo simulations (10,000 replicates). (*D*) Annual mean surface air temperature (SAT) at Irkutsk (World Meteorological Organization station 30710 [52°16′20″ N, 104°18′29″ E; elevation, 467 m]) (Fig. 1). (*E*) South basin annual ice cover duration. The black lines and gray confidence intervals on individual panels show a GAM fitted to each time series.

In deep lakes across the world, thermobaric convection is resistant to direct surface water warming from anthropogenic climate change (26). Lake Baikal is no exception to this with deep ventilation from coastal downwelling believed to be resilient to past and predicted future changes in surface water temperature (SWT) (27–29) (Fig. 3*A*). Instead, deep ventilation in Lake Baikal is predominantly controlled by wind, through generating Ekman transport toward the coast, which is able to trigger thermobaric instability in the water column and thus deep coastal downwellings (9, 10, 25, 28, 30, 31). Typically occurring twice a year, when the lake is weakly inversely stratified in December/January before ice formation on the lake and after ice-out in late spring (May/June), this ventilation results in 12.5% of the deep water layer being renewed (10 to 100 km^3^/y) and the persistence of a fully oxygenated water column (10, 25, 31).

Through the 20th and 21st centuries, climate reanalysis data show a clear increase in wind strength over Lake Baikal during the winter and late spring period in which coastal ventilation occurs (Fig. 3*B*). Over the same interval, modeled rates of Ekman transport also increase (Fig. 3*B*). With the increases in wind strength and Ekman transport both concordant with reconstructed changes in nutrient supply, we attribute the 20th- and 21st-century photic zone increases in silicic acid to a marked increase in wind-driven Ekman transport and so deep ventilation in Lake Baikal (Fig. 3). It is unclear to what extent increase in wind strength over Lake Baikal can be associated with wider global climate changes (34). However, regional climate change over the last 100 y is known to have reduced annual ice cover duration in response to rising surface air temperatures (SATs) (35, 36) (Fig. 3 *A*, *D*, and *E*). While such a change is unlikely to have directly impacted deep water ventilation in Lake Baikal (25), research on Lake Superior has demonstrated that increasing SAT and shortening of the ice season both warmed SWT and destabilized the atmospheric boundary layer, increasing wind speeds above the lake (37). With winds over Lake Baikal chiefly controlled by local pressure phenomena due to differential heating between land and water (10), similar SAT, SWT, and ice cover changes in Lake Baikal may be triggering the same process over Lake Baikal, altering lake wind conditions and deep ventilation (Fig. 3). Reduced ice cover and changing SWT dynamics may also increase the period of time favorable for deep ventilation, which occurs when SWTs are below 4 °C and close to water temperatures at the lake bottom.

Rivers in the catchment (>350 rivers; ∼540,000 km^2^) also supply nutrients to the lake, but due to the long residence time of both waters and nutrients in Lake Baikal these inputs represent only a fraction of all nutrients annually cycled within the lake (23). While deteriorations in river water quality have been observed (38), we have found no evidence that anthropogenic activity has altered the silicic acid concentrations or δ^30^Si composition of waters flowing into the lake, based on spatial and temporal river measurement across the Selenga River drainage basin that supplies 62% of all riverine inputs to Lake Baikal (21) (Fig. 1). With instrumental records also showing interdecadal variability but no long-term change in the flow of rivers draining into Lake Baikal through the 20th and 21st centuries (39), it is unlikely that natural or anthropogenic alterations to the catchment have increased the supply of silicic acid to Lake Baikal. Similarly, while anthropogenic activity from urbanization, mining, and deforestation in the immediate vicinity of the lake has resulted in significant volumes of wastewater entering the lake, and even shoreline eutrophication (11, 12), this development has largely occurred since the 1960s and so is incapable of initiating the increase in silicic acid supply from the mid-19th century onward. Therefore, we conclude that changes in deep ventilation within Lake Baikal are the principal mechanism to explain increases in photic zone nutrient supply during the 20th and 21st centuries.

### Ecological Implications.

**Fig. 4.**
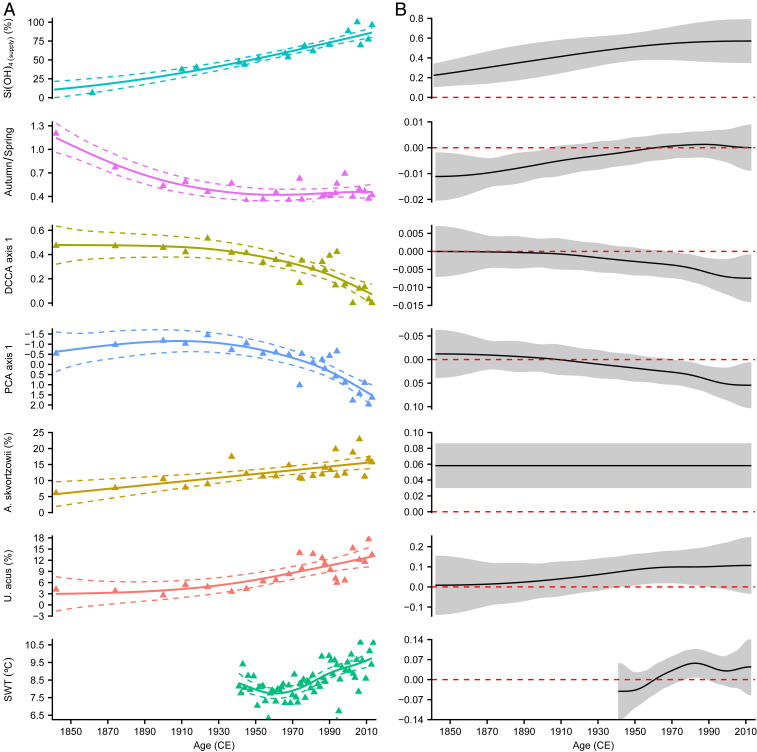
Diatom community changes at core site BAIK13-7 (Fig. 1) (14, 16). (*A*) Changes in photic zone silicic acid supply (relative to a value of 100% at 2005 CE) together with the ratio of autumn/spring taxa, detrended canonical correspondence analysis (DCCA) axis 1 scores reflecting diatom composition turnover, principal-components analysis (PCA) axis 1 scores reflecting changing taxa composition, relative abundance of *U. acus* and *A. skvortzowii*, as well as mean surface water temperatures (SWTs) (May to October) from shoreline locations across Lake Baikal (29). (*B*) First derivatives and 95% simultaneous confidence intervals of GAMs fitted to each time series. Where the simultaneous interval does not include 0, the models detect significant temporal change in the proxy record.

Lake Baikal’s biodiversity and ecosystem functioning has been subject to abrupt and rapid change through the Holocene, including changes to carbon dynamics (40) and endemic diatom communities (22, 41). With pelagic communities in the lake known for their ability to rapidly respond to changing conditions (42), there is a need to assess the resilience of Lake Baikal and its high endemicity to anthropogenic forcings including changes in biogeochemical cycling. Increases in both endemic and nonendemic diatom taxa are observed from the end of the LIA in response to warmer climate conditions and reduced snow/ice cover on the lake, increasing turbulent mixing, light availability, and changes to the suspension of diatoms in the photic zone (16, 43). Simultaneous with this and the higher rates of Ekman transport driven nutrient supply to the photic zone from the late 19th century, are significant relative decreases (increases) in autumn (spring) productivity, driven by declines in endemic taxa including *Crateriportula inconspicua* and *Cyclotella minuta* (Fig. 4).

Changes to the diatom community and compositional turnover—the change in species composition and relative abundances—continue through the 20th and 21st centuries (*P* = 0.004), with breakpoint analyses and generalized additive models (GAMs) indicating a significant escalation from the 1970s onward (Fig. 4). Community changes in Lake Baikal in the second half of the 20th century and the expansion and displacement of endemic taxa have previously been attributed to increased SWT and summer thermal stratification (14, 16) (Fig. 4). While it remains unclear to what extent these changes can also be directly attributed to the concordant increase in photic zone nutrient supply, redundancy analysis suggests that nutrient supply may account for almost one-quarter of diatom community variation exhibited. Furthermore, changes in community turnover are strongly associated with relative abundance increases in the endemic *Aulacoseira skvortzowii* (*r* = −0.68, *P* < 0.005) and cosmopolitan *Ulnaria acus* (*r* = −0.83; *P* < 0.005), trends that are mirrored elsewhere across the lake (14, 16, 22, 44) (Fig. 4). *U. acus* is a taxa associated with higher dissolved silica concentrations in the water column (22, 41), while cells of both *A. skvortzowii* and *U. acus* require strong winds/currents to be transported from their littoral region habitats to colonize pelagic waters (45). Consequently, increasingly deterministic processes (46) linked to high nutrient (silicic acid) supply and wind-driven Ekman transport, as well as warmer SWT, may be driving shifts in compositional turnover in Lake Baikal, especially during the past 50 y.

The potential impact of Ekman transport-driven nutrient supply on the diatom community highlights the ability for changes in deep ventilation to also increase other forms of primary productivity, reduce relative rates of nutrient utilization, and alter food–web interactions and nutrient resources in Lake Baikal (15). This, in turn, has the capability to exacerbate problems caused by the emergence of anthropogenic stressors through the 20th and 21st centuries. Climate change, primarily through reduced ice cover and higher SWT, as well as shoreline eutrophication has impacted a range of autotrophic and heterotrophic organisms in littoral and pelagic regions across the lake (11–14, 42, 44). Future anthropogenic warming in the 21st century is predicted to shift primary productivity to less silicified littoral diatoms and autotrophic picoplankton (15, 16, 22, 44, 47). While the combined impact of these changes on energy transfers and trophic cascades in Lake Baikal’s endemic ecosystem remains unresolved (19, 42), it is clear that as SWTs have increased, heavier pelagic diatoms are living at deeper depths in the photic zone, concurrent with upward shifts in many groups of zooplankton consumers, leading to an alteration in the spatial overlap between the grazers and their food (42).

Our results show that the 20th and 21st centuries have been characterized by a significant increase in deep ventilation in Lake Baikal, which increased the rates at which deep water nutrients are supplied to the photic zone. Combined with models showing the susceptibility of coastal downwelling to changes in wind strength (28), these findings highlight the need for robust estimates of future wind changes over Lake Baikal under Intergovernmental Panel on Climate Change Representative Concentration Pathway/Shared Socioeconomic Pathway scenarios. This is key to determining the vulnerability of Lake Baikal to future physicochemical alterations as well as aiding ongoing efforts to understand ecosystem functions in this World Heritage Site. Wind characteristics over Lake Baikal are complex and hard to predict (28), with recent observations outside the main winter ventilation season showing reductions in wind strength (29) rather than the increase forecast to occur with anthropogenic climate change (10). However, with indications that anthropogenic warming will continue to reduce annual ice cover over the lake (35, 36), further increases in wind activity over the lake in response to higher SWT and a destabilized atmospheric boundary layer seem assured (37). Together, these changes risk increasing turbulent mixing and deep ventilation-driven nutrient supply to the photic zone. Alongside shoreline eutrophication, this threatens the balance between endemic and cosmopolitan species across both littoral and pelagic regions of the lake (13, 22, 44), impacting feeding strategies of at least the lake’s primary consumers (42).

## Materials and Methods

### Methods.

#### Study site.

Sediment cores from site BAIK13-1 (51°46′04.2″N, 104°24′58.6″E; water depth, 1,360 m) and BAIK13-4 (51°41′33.8″N, 104°18′00.1″E; water depth, 1,360 m) were collected from the south basin of Lake Baikal in March 2013 using a UWITEC corer with PVC-liners (Ø 63 mm) that enabled undisturbed recovery of material at the sediment/water interface (Fig. 1). Cored through ∼78 to 90 cm of ice, cores BAIK13-1C (50 cm) and BAIK13-4F (33 cm) were subsampled in the field at a resolution of 0.2 cm and transported to the United Kingdom. Both sites are located >120 km away from the Selenga Delta and other major river inflows/sources of nutrients to the lake (Fig. 1).

#### Chronologies.

Well-constrained ^210^Pb-derived age models for core BAIK13-1C and BAIK13-4F have previously been published covering the last ∼150 y (48). To extend these age models to cover samples prior to 1850 CE, radiocarbon (^14^C) dating was completed on two samples at BAIK13-1C and one sample at BAIK13-4F. All ^210^Pb and calibrated ^14^C dates were then combined to create a new Bayesian radiocarbon age model for each site using Bacon (*SI Appendix*).

#### Isotope analysis.

Diatoms were extracted for isotope analysis with a combination of 5% HCl and 30% H_2_O_2_, alongside sodium polytungstate heavy liquid separation at specific gravities of ∼2.2 g/mL, used to remove nondiatom contaminants (48). All samples were screened using a Zeiss Axiovert 40 C inverted microscope, scanning electron microscope, and X-ray fluorescence to confirm sample purity and the absence of nondiatom contaminants. Only samples with an Al:Si contamination <1% and that visibly demonstrated diatom-rich assemblages were analyzed. This quality control ultimately limits the final number of analyzed samples and so the resolution of the isotope record. Diatoms in the analyzed samples are dominated by planktonic taxa including *Aulacoseira baicalensis*, *Aulacoseira skvortzowii*, *Crateriportula inconspicua*, *Cyclotella minuta*, *Stephanodiscus meyerii*, and *Ulnaria acus*. Given the ecology of these species, our isotope record is interpreted as reflecting mean annual conditions within the photic zone in Lake Baikal, with a small bias toward spring months when diatom productivity peaks (47). Due to their close proximity and the strong age models for both sites, samples from each core are combined together to create a composite record for the south basin of Lake Baikal.

Alkaline fusion (NaOH) of cleaned diatom samples and subsequent cation exchange (Bio-Rad; AG50W-X12) followed existing methodologies (20). Samples were analyzed in wet-plasma mode using the high mass-resolution capability of a Thermo Fisher Neptune Plus MC-ICP-MS (multicollector inductively coupled plasma mass spectrometer) at the British Geological Survey. Full analytical methods, including practices applied to minimize instrument induced mass bias and drift, are detailed in refs. 20, 21. Full procedural blank compositions from MC-ICP-MS analyses were ∼48 ng compared to typical sample amounts of ∼3,900 ng. Using the worst-case scenario (i.e., calculated using the sample with the lowest Si concentration), this level of blank could result in a potential shift in sample composition by <0.03‰, which was insignificant relative to the typical <0.11‰ propagated sample uncertainties. All uncertainties are reported as 2σ absolute, and incorporate an excess variance derived from repeat analysis of the NBS 28 reference material, which was quadratically added to the analytical uncertainty of each measurement. Long-term (∼2 y) reproducibility and machine accuracy are assessed via the analysis of the Diatomite secondary reference material, with data (+1.24‰ ± 0.18‰, 2σ, *n* = 244) agreeing well with the published consensus value (+1.26‰ ± 0.2‰, 2σ) (49).

#### BSi.

In line with previous work on Lake Baikal, BSi concentrations were measured on samples from BAIK13-1C at 0.2-cm resolution using a single-step wet-alkaline digestion technique. Following digestion of 30 mg of freeze-dried sediment in a weak (1% Na_2_CO_3_) solution, designed to minimize dissolution of aluminosilicates, aliquots were taken after 5 h and analyzed for dissolved silica using colorimetric determination. Replicate analyses of sample material indicated an analytical reproducibility of 0.49%. BSi MARs were then calculated using dry bulk density and sediment accumulation rates for the core.

#### Silicic acid utilization/supply.

Lake Baikal is best characterized by an open system model (21) in which records of δ^30^Si_diatom_ are a function of the isotope composition of dissolved silicic acid [δ^30^Si(OH)_4_] supplied to the photic zone (δ^30^Si_lake_), the fraction of Si(OH)_4_ remaining in the water (*f*), and the enrichment factor between diatoms and silicic acid (*ε*):

$$\delta^{30}{\text{Si}}_{\text{diatom}}=\delta^{30}{\text{Si}}_{\text{lake}}+\varepsilon \;•\;f.$$ [1]

From this, changes in the relative rate of photic zone silicic acid utilization (i.e., 1 − *f*) can be obtained given that δ^30^Si_lake_ and *ε* have been constrained in Lake Baikal at 1.71‰ and −1.61‰, respectively (20, 21):

$$\text{Si}{\left(\text{OH}\right)}_{4\left(\text{utilisation}\right)}=\left(1-\frac{\delta^{30}{\text{Si}}_{\text{diatom}}-1.71}{1.61}\right)\;•\;100.$$ [2]

While changes in silicic acid utilization will mirror changes in δ^30^Si_diatom_ (Fig. 2 *A* and *B*), BSi MAR can be used as a measure of siliceous productivity to account for the fact that variations in relative rates of nutrient utilization can occur due to changes in the following: 1) siliceous productivity; and/or 2) the rate of nutrient supply to the photic zone, altering *f* in Eq. **1**. With this, changes in the supply of Si(OH)_4_ to the photic zone are calculated relative to the sample at 2005 CE:

$$\text{Si}{\left(\text{OH}\right)}_{4\left(\text{supply}\right)}=\frac{{\text{BSi MAR}}_{\text{sample}}/{\text{BSi MAR}}_{2005\text{CE}}}{\text{Si}{\left(\text{OH}\right)}_{4\left(\text{utilisation}\right)}/\text{Si}{\left(\text{OH}\right)}_{4\left(\text{utilisation}-2005\text{CE}\right)}}.$$ [3]

#### Ekman transport.

Coastal deep ventilation in Lake Baikal is generated by winds aligned with the main axis of the lake, producing a net transport of surface water toward the lake coast to the right of the wind direction. This phenomenon, known as Ekman transport, is caused by the planetary rotation and is defined as follows:

$$M=\frac{\tau }{f\;•\;\rho },$$ [4]

where *f* is the Coriolis frequency, *ρ* is the density of water, and *τ* is the wind shear stress:

$$\tau =\rho_{a}C_{D}W^{2},$$ [5]

in which *ρ*_*a*_ is the density of air, *C*_*D*_ is the drag coefficient, and *W* is the wind speed component parallel to Lake Baikal's coast, which on average has a counterclockwise angle of ∼50° relative to the horizontal direction. Previous work has shown that winds from the northeast are the most favorable to generate Ekman transport and subsequent deep ventilation in Lake Baikal, due to their predominance and high speed as well as the bathymetry of the lake with steeper slopes at the northwest shore where these winds produce coastal downwelling (24, 29). Using CERA-20C (32) (resolution, 125 km) and ERA5 (33) (resolution, 30 km) wind reanalysis data (height, 10 m) around the barycentre of the Lake Baikal (53.375°N, 108.125°E), Ekman transport generated by winds from northeast were evaluated in the months when deep ventilation occurs in Lake Baikal (May to June and December to January) and is used as a quantitative measure of the potential intensity of deep ventilation. Specifically, as persistent wind events are required to generate coastal downwelling, for each year (*i*) and downwelling season (*s*), the cumulative Ekman transport ($M_{c}$) is calculated across periods when the wind both blows from northeast and is interrupted by winds from other directions for less than 1 d, with values then averaged over each year and downwelling season [$\bar{M_{c}}\left(i,s\right)$, where the overbar indicates averaging]. Ekman transport anomalies of a given year and downwelling season can then be calculated relative to the mean Ekman transport from 1990 to 2000 CE (*SI Appendix*):

$$\text{Anomaly}\bar{M_{c}}\left(i,s\right)=\frac{\bar{M_{c}}\left(i,s\right)}{\bar{M_{c}}\left(1990-2000,s\right)}.$$ [6]

#### Statistical analyses.

Data normality was checked using a Shapiro–Wilk test with comparisons between datasets then performed using either a Wilcoxon rank sum test or *t* test. The uncertainty associated the variables in calculating silicic acid utilization, BSi MAR, and silicic supply (Eq. **3**) was calculated assuming a normal distribution for proxy data uncertainty and Monte Carlo simulations (10,000 replicates) (*SI Appendix*).

The magnitude of diatom turnover at site Baik13-7 (Fig. 1) was estimated using detrended correspondence analysis, with square root transformation of the species data to stabilize variance (*SI Appendix*). The axis 1 gradient length was 0.652 SD units, so data were reanalysed using principal-components analysis (PCA). Diatom compositional turnover through time was estimated by constraining relative abundance data with sample dates derived from ^210^Pb radiometric dating. Compositional turnover was calculated using detrended canonical correspondence analysis (DCCA), with a square root transformation of diatom relative abundance taxa (*SI Appendix*). The degree to which nutrient supply may influence diatom community composition was estimated using redundancy analysis, with time partialled out as a covariable.

GAMs were calculated with restricted maximum-likelihood smoothness selection. To account for temporal autocorrelation, all models included a continuous-time first-order autoregressive [CAR(1)] process. Intervals of significant temporal change in a GAM were detected using the first-order derivative of the fitted trend and a 95% simultaneous confidence interval (50) (*SI Appendix*).

## Supplementary Material

Supplementary File

Supplementary File

Supplementary File

Supplementary File

Supplementary File

## Data Availability

All study data are included in the article and *SI Appendix*.

## References

[1] C. M. O’Reilly., Rapid and highly variable warming of lake surface waters around the globe. Geophys. Res. Lett. 42, 10773–10781 (2015).

[2] G. Grill., Mapping the world’s free-flowing rivers. Nature 569, 215–221 (2019). 31068722 10.1038/s41586-019-1111-9

[3] D. Obrist., A review of global environmental mercury processes in response to human and natural perturbations: Changes of emissions, climate, and land use. Ambio 47, 116–140 (2018). 29388126 10.1007/s13280-017-1004-9 PMC5794683

[4] S. R. Hall, E. L. Mills, Exotic species in large lakes of the world. Aquat. Ecosyst. Health Manage. 3, 105–135 (2000).

[5] Y. Vadeboncoeur, P. B. McIntyre, M. J. V. Zanden, Borders of biodiversity: Life at the edge of the world’s large lakes. Bioscience 61, 526–537 (2011).

[6] IPBES, The IPBES Assessment Report on Land Degradation and Restoration, L. Montanarella, R. Scholes, A. Brainich, Eds. (Secretariat of the Intergovernmental Science-Policy Platform on Biodiversity and Ecosystem Services, Bonn, Germany, 2018).

[7] S. E. Hampton., Recent ecological change in ancient lakes. Limnol. Oceanogr. 63, 2277–2304 (2018).

[8] O. A. Timoshkin, “Biodiversity of Baikal fauna: State-of-the-art (preliminary analysis)” in *New Scope on the Boreal Ecosystems in East Siberia, Proceedings of the International Symposium DIWPA Ser. No. 2*, E. Wada, O.A. Timoshkin, N. Fujita, K. Tanida, Eds. (The Siberian Branch of the Russian Academy of Sciences Press, Novosibirsk, 1997), pp. 35–76.

[9] R. F. Weiss, E. C. Carmak, V. M. Koropalov, Deep-water renewal and biological production in Lake Baikal. Nature 349, 665–669 (1991).

[10] M. N. Shimaraev, V. I. Verbolov, N. G. Granin, P. P. Sherstyankin, Physical Limnology of Lake Baikal: A Review, (BICER, Irkutsk, Okayama, 1994).

[11] L. S. Kravtsova., Nearshore benthic blooms of filamentous green algae in Lake Baikal. J. Great Lakes Res. 40, 441–448 (2014).

[12] O. A. Timoshkin., Rapid ecological change in the coastal zone of Lake Baikal (East Siberia): Is the site of the world’s greatest freshwater biodiversity in danger? J. Great Lakes Res. 42, 487–497 (2016).

[13] L. R. Izmest’eva., Lake-wide physical and biological trends associated with warming in Lake Baikal. J. Great Lakes Res. 42, 6–17 (2016).

[14] S. L. Roberts., Diatom evidence of 20th century ecosystem change in Lake Baikal, Siberia. PLoS One 13, e0208765 (2018). 30566423 10.1371/journal.pone.0208765 PMC6300214

[15] M. V. Moore., Climate change and the World’s “Sacred Sea”–Lake Baikal, Siberia. Bioscience 49, 405–417 (2009).

[16] A. W. Mackay., Diatom succession trends in recent sediments from Lake Baikal and their relation to atmospheric pollution and to climate change. Philos. Trans. R. Soc. Lond., B 353, 1011–1055 (1998).

[17] L. A. Barboza.; PAGES 2k Consortium, Consistent multidecadal variability in global temperature reconstructions and simulations over the Common Era. Nat. Geosci. 12, 643–649 (2019). 31372180 10.1038/s41561-019-0400-0 PMC6675609

[18] N. G. Granin., Turbulent mixing under ice and the growth of diatoms in Lake Baikal. Verh. Int. Ver. Theor. Angew. Limnol. 27, 2812–2814 (2000).

[19] M. V. Moore, Trophic coupling of the microbial and the classical food web in Lake Baikal, Siberia. Freshw. Biol. 64, 138–151 (2019).

[20] V. N. Panizzo., Insights into the transfer of silicon isotopes into the sediment record. Biogeosciences 13, 147–157 (2016).

[21] V. N. Panizzo., Constraining modern day silicon cycling in Lake Baikal. Global Biogeochem. Cycles 31, 556–574 (2017).

[22] A. W. Mackay, D. B. Ryves, D. W. Morley, D. H. Jewson, P. Rioual, Assessing the vulnerability of endemic diatom species in Lake Baikal to predicted future climate change: A multivariate approach. Glob. Change Biol. 12, 2297–2315 (2006).

[23] B. Müller., Internal carbon and nutrient cycling in Lake Baikal: Sedimentation, upwelling, and early diagenesis. Global Planet. Change 46, 101–124 (2005).

[24] E. Troitskaya., Cyclonic circulation and upwelling in Lake Baikal. Aquat. Sci. 77, 171–182 (2015).

[25] M. Schmid., Lake Baikal deepwater renewal mystery solved. Geophys. Res. Lett. 35, L09605 (2008).

[26] B. Boehrer, R. Fukuyama, K. Chikita, Stratification of very deep, thermally stratified lakes. Geophys. Res. Lett. 35, L16405 (2008).

[27] S. E. Hampton., Sixty years of environmental change in the world’s largest freshwater lake—Lake Baikal, Siberia. Glob. Change Biol. 14, 1947–1958 (2008).

[28] S. Piccolroaz, M. Toffolon, The fate of Lake Baikal: How climate change may alter deep ventilation in the largest lake on Earth. Clim. Change 150, 181–194 (2018).

[29] T. G. Potemkina, V. L. Potemkin, A. L. Fedotov, Climatic factors as risks of recent ecological changes in the shallow zone of Lake Baikal. Russ. Geol. Geophys. 59, 556–565 (2018).

[30] C. Tsimitri., Drivers of deep-water renewal events observed over 13 years in the South Basin of Lake Baikal. J. Geophys. Res. Oceans 120, 1508–1526 (2015).

[31] S. Piccolroaz, M. Toffolon, Deep water renewal in Lake Baikal: A model for long‐term analyses. J. Geophys. Res. Oceans 118, 6717–6733 (2013).

[32] P. Laloyaux., CERA‐20C: A coupled reanalysis of the twentieth century. J. Adv. Model. Earth Syst. 10, 1172–1195 (2018).

[33] Copernicus Climate Change Service, ERA5: Fifth generation of ECMWF atmospheric reanalyses of the global climate (Copernicus Climate Change Service Climate Data Store [CDS], 2017). https://cds.climate.copernicus.eu/cdsapp#!/home. Accessed 31 October 2019.

[34] Z. Zeng., A reversal in global terrestrial stilling and its implications for wind energy production. Nat. Clim. Chang. 9, 979–985 (2019).

[35] M. N. Shimaraev, L. N. Kuimova, V. N. Sinyukovich, V. V. Tsekhanovskii, Manifestation of global climatic changes in Lake Baikal during the 20th century. Dokl. Earth Sci. 383, 288–291 (2002).

[36] M. C. Todd, A. W. Mackay, Large scale climatic controls on Lake Baikal ice cover. J. Climatol. 16, 3186–3199 (2003).

[37] A. Desai, J. Austin, V. Bennington, G. A. McKinley, Stronger winds over a large lake in response to weakening air-to-lake temperature gradient. Nat. Geosci. 2, 855–858 (2009).

[38] L. M. Sorokovikova, V. N. Sinyukovich, I. V. Tomberg, I. I. Marinaite, T. V. Khodzher, Assessing the water quality in the tributary streams of Lake Baikal from chemical parameters. Geogr. Nat. Resour. 36, 31–39 (2015).

[39] T. G. Potemkina, V. L. Potemkin, Sediment load of the main rivers of Lake Baikal in a changing environment (east Siberia, Russia). Quat. Int. 380-381, 342–349 (2015).

[40] A. W. Mackay., Holocene carbon dynamics at the forest-steppe ecotone of southern Siberia. Glob. Change Biol. 23, 1942–1960 (2017). 10.1111/gcb.13583 PMC6849524 27935187

[41] J. P. Bradbury., A synthesis of post-glacial diatom records from Lake Baikal. J. Paleolimnol. 10, 213–252 (1994).

[42] S. E. Hampton, D. K. Gray, L. R. Izmest’eva, M. V. Moore, T. Ozersky, The rise and fall of plankton: Long-term changes in the vertical distribution of algae and grazers in Lake Baikal, Siberia. PLoS One 9, e88920 (2014). 24586441 10.1371/journal.pone.0088920 PMC3934874

[43] A. W. Mackay., 1000 years of climate variability in central Asia: Assessing the evidence using Lake Baikal (Russia) diatom assemblages and the application of a diatom-inferred model of snow cover on the Lake. Global Planet. Change 46, 281–297 (2005).

[44] N. A. Bondarenko., Recent changes in the spring microplankton of Lake Baikal, Russia. Limnologica 75, 19–29 (2019).

[45] D. H. Jewson., Resting stages and ecology of the planktonic diatom *Aulacoseira skvortzowii* in Lake Baikal. Limnol. Oceanogr. 53, 1125–1136 (2008).

[46] C. A. Larson, L. Adumatioge, S. I. Passy, The number of limiting resources in the environment controls the temporal diversity patterns in the algal benthos. Microb. Ecol. 72, 64–69 (2016). 26943146 10.1007/s00248-016-0741-9

[47] G. I. Popovskaya, Ecological monitoring of phytoplankton in Lake Baikal. Aquat. Ecosyst. Health Manage. 3, 215–225 (2000).

[48] G. E. A. Swann., Lake Baikal isotope records of Holocene Central Asian precipitation. Quat. Sci. Rev. 189, 210–222 (2018).

[49] B. C. Reynolds., An inter-laboratory comparison of Si isotope reference materials. J. Anal. At. Spectrom. 22, 561–568 (2007).

[50] G. L. Simpson, Modelling palaeoecological time series using generalised additive models. Front. Ecol. Evol. 6, 149 (2018).

