# Supplementary material for: Changing nutrient cycling in Lake Baikal, the world’s oldest lake
Source: Proc Natl Acad Sci U S A. 2020 Oct 19;117(44):27211–7. doi: 10.1073/pnas.2013181117 (PMC7959572; doi:10.1073/pnas.2013181117)
Supplement: Supplementary File [file pnas.2013181117.sapp.pdf]

Supplementary Information for

Changing nutrient cycling in Lake Baikal, the world's oldest lake

George E. A. Swann, Virginia N. Panizzo, Sebastiano Piccolroaz, Vanessa Pashley, Matthew S. A. Horstwood, Sarah Roberts, Elena Vologina, Natalia Piotrowska, Michael Sturm, Andre Zhdanov, Nikolay Granin, Charlotte Norman, Suzanne McGowan, Anson W. Mackay

Paste corresponding author name here
Email: george.swann@nottingham.ac.uk, virginia.panizzo@nottingham.ac.uk

This PDF file includes:

Supplementary text
Figures S1 to S8
Table S1
Legends for Datasets S1 to S4
SI References

Other supplementary materials for this manuscript include the following:

Datasets S1 to S4

www.pnas.org/cgi/doi/10.1073/pnas.2013181117

Supplementary Information Text

Lake Baikal

Lake Baikal is the world's oldest lake, lying in a rift zone in south eastern Siberia (103°43'-109°58'E, 51°28'-55°47'N; Fig. 1) that began to form over 30 million years ago. Divided into three basins (south, central and north) (Fig. 1), waters in the lake are primarily derived from direct precipitation (16.3%) and riverine inputs (83.2%), with >350 rivers draining a catchment (c. 540,000 km^2) that extends into Mongolia and supports over 3 million people[1]. A key feature of Lake Baikal is the high degree of biodiversity with over 2,500 flora and fauna, the majority of which are endemic[2]. Such high levels of endemicity have been attributed to the lake's age and to a fully oxygenated water column, driven by seasonal overturning and deep-water renewal[3]. Cited as the "most outstanding example of a freshwater ecosystem" this, together with its high level of endemicity, led the site being designated a World Heritage Site in 1996[4]. As a result of Lake Baikal's high-profile ecosystem, in addition to its historic and cultural status as a sacred site for indigenous Buryat people, the lake is of interest to a range of national/international agencies (e.g., UNESCO, World Bank and the UN Integrated Natural Resource Management in the Baikal Basin Transboundary Ecosystem) as well as organisations and academic institutions (e.g., Rivers without Boundaries and the Limnological Institute Siberian Branch of the Russian Academy of Sciences) supporting biodiversity/conservation and other ecosystem services.

Today, Lake Baikal is responding strongly to anthropogenic pressures that are threatening the stability of its ecosystem. Whilst the Baikalsk Pulp and Paper Mill (BPPM) (operating 1966-2008 and 2010-2013) on the edge of Lake Baikal was the most notorious source of direct pollution, other hydrologically-derived contamination originating from major conurbations, industrial centres, mining and agricultural practises has leading to shoreline eutrophication and rising mercury concentrations[5-9]. Anthropogenic climate change is also increasing water column temperature and reducing ice cover duration/thickness[10-15]. Together, these changes are impacting or predicted to impact existing balances between endemic and cosmopolitan biota[14,16-19] and food-web interactions inextricably linked to ice dynamics and seasonal overturning, from the rapid growth of primary producers underneath ice to the lake's top consumer, *Phoca* or *Pusa sibirica* (endemic freshwater seal)[20,21]. In order to ensure that future development and policy plans for the catchment are capable of sustaining Lake Baikal's unique ecosystem, there is a need to not only understand the impact of anthropogenic influence on the lake, but to also distinguish between natural and non-natural variability in the lake's ecosystem by placing measurements into a historical context.

Diatom productivity

Diatoms are unicellular siliceous algae that dominate primary productivity in many aquatic systems across the globe, where they photosynthesise within the photic zone in the uppermost section of the water column. Within Lake Baikal, diatoms form a key component of phytoplankton[22-26], with upwards of 40% of taxa endemic to the lake[27-29]. Due to their siliceous frustule (shell), which helps protect them from dissolution, diatoms also dominate the fossil assemblage, forming c. 98% of all biogenic silica (BSi) in Lake Baikal's sediments[30]. Existing palaeoenvironmental research, from Lake Baikal's uninterrupted sediment record spanning the past c. 20 million years[31], has often focused on diatoms, taking advantage of the strong linkages between individual species and environmental conditions[32].

There are two main seasonal diatom blooms in Lake Baikal, with the biomineralisation of dissolved silica (DSi; i.e., silicic acid [$SiOH_4$]) dominating silica cycling in the lake[33,34] (Fig. S1). Whilst diatoms annually export 1,830 mmol m^{-2} of DSi from the photic zone to deep waters, this can increase to 2,700 mmol m^{-2} during "Melosira years" in which exceptionally large blooms of the endemic diatom *Aulacoseira baicalensis* occur[34]. Each year the first diatom bloom starts under ice in spring, with the greatest rate of diatom growth occurring after ice break-up when frustules undergo rapid cell division in response to increases in light availability and turbulent mixing in the uppermost sections of the water column[35,36]. During this period, diatoms can

constitute 50-90% of phytoplankton biomass in the lake and consume up to 90% of available silicic acid in the photic zone[25,37]. Following atmospheric warming of the surface waters a summer stratification develops, which leads to low surface water nutrient conditions and to diatoms being replaced by pico-cyanobacteria as the dominant phytoplankton[23,35,38]. As summer stratification breaks down in autumn, the return of turbulent mixing triggers a second diatom bloom that is notably smaller than the spring bloom in terms of magnitude and silicic acid utilisation[35,37]. Today, productivity in the photic zone of Lake Baikal is co-limited by nitrogen and phosphorus[39,40]. Therefore, increased upwelling of deep water rich in silicon, nitrogen and phosphate to the photic zone (630 mmol SiO_2 m^{-2} $year^{-1}$; 93 mmol NO_3^- m^{-2} $year^{-1}$; 5 mmol P m^{-2} $year^{-1}$)[34] will increase both biological productivity and rates of silicic acid utilisation.

Silicon exported by diatoms from the photic zone will, depending on rates of dissolution and other sedimentation processes, either be remineralised in deep waters or transferred into the sediment record. Combined with a long silicon residence time in Lake Baikal of 100-170 years, this results in deep water nutrient concentrations for nitrate, phosphate and silicate that are significantly higher than those at the surface[34,41-44]. The mixing of these waters back to the photic zone, therefore, provides an essential source of nutrients to fuel further primary productivity. This internal cycling of regenerated silicon back to the photic zone significantly exceeds riverine inputs to the lake of 312 mmol m^{-2} yr^{-1} [33,34], with riverine inputs quickly diluted by pre-existing lake waters[37]. Consequently, changes in the rate at which these nutrient-rich deep waters are mixed into the photic zone have the potential to significantly alter biogeochemical cycling, aquatic productivity and food-web interactions in Lake Baikal.

Previous studies have argued that silicic acid concentrations have decreased throughout Lake Baikal's water column from 1995-2001 CE, in response to an increased export of silicon by diatoms into the sediment record[44,45]. These measurements, however, are potentially biased by sampling, which varies in any given year from May to September and so is influenced by aforementioned seasonal/monthly variations in biological productivity and nutrient cycling to the photic zone. The observation of a decline in Lake Baikal silicic acid concentrations is also not in agreement with nutrient mass-balance calculations for the lake[34]. Within the $\delta^{30}Si_{diatom}$ record presented in this study, there are insufficient samples which allow further investigation of the reported decline in silicic acid concentrations from 1995-2001 CE[44,45], although our reconstructed record indicates a moderate increase in rates of silicic acid supply to the photic zone between two samples dated to 1990 CE and 2000 CE respectively.

Silicon isotopes

Silicon has three stable isotopes: ^{28}Si (92.2% relative abundance on Earth), ^{29}Si (4.7%), and ^{30}Si (3.1%)[46]. The silicon isotope ratios of diatoms ($\delta^{30}Si_{diatom}$) are expressed as δ and reported in part per mille (‰):

$$\delta^{30}\mathrm{Si}_{\mathrm{diatom}} = \frac{\mathrm{R}_{\mathrm{diatom}} - \mathrm{R}_{\mathrm{reference}}}{\mathrm{R}_{\mathrm{reference}}} \bullet 1000 \qquad (1)$$

where R is $^{30}Si/^{28}Si$ and "reference" refers to NBS-28: the standard reference material for silicon. There is a well constrained mass dependent fractionation between $\delta^{30}Si$ ($^{30}Si/^{28}Si$) and $\delta^{29}Si$ ($^{29}Si/^{28}Si$) such that:

$$\delta^{30}\mathrm{Si} = 1.96 \bullet \delta^{29}\mathrm{Si} \qquad (2)$$

During biomineralisation, diatoms preferentially uptake the lighter ^{28}Si isotope over the heavier ^{29}Si and ^{30}Si from silicic acid in the water column. This results in a progressive enrichment of ^{30}Si in both the dissolved and particulate phases within the photic zone, so that $\delta^{30}\mathrm{Si}_{\mathrm{diatom}}$ can be used to infer changes in the rate of silicic acid utilization. The magnitude of this isotopic fractionation between diatoms and dissolved silicic acid is defined by (α):

$$\alpha_{\text{diatom-DSi}} = \frac{{}^{30}\text{Si(OH)}_4/{}^{28}\text{Si(OH)}_4}{{}^{30}\text{Si}_{\text{diatom}}/{}^{28}\text{Si}_{\text{diatom}}} \qquad (3)$$

Most commonly, this isotopic fractionation is defined in terms of the enrichment factor ε:

$$\varepsilon \cong 1000 \bullet (\alpha_{\text{diatom-DSi}} - 1) \qquad (4)$$

Whilst marine diatoms grown under laboratory conditions have shown evidence of species-dependent silicon isotope fractionation[47], such a process has not been demonstrated in lacustrine diatoms/systems. Instead, previous work analysing diatoms in sediment traps through Lake Baikal's water column has constrained ε at −1.61‰[48]. Reassessment of the raw data, assuming a normal distribution for proxy data uncertainty and using 10,000 replicate Monte Carlo simulations with the Monte Carlo package in R[49,50] indicates an uncertainty for ε of 0.11‰ (1σ). Given this, and using the Rayleigh fractionation model to describe changes in the isotopic composition of $\delta^{30}Si_{diatom}$ and $\delta^{30}Si(OH)_4$ [51], measurements of $\delta^{30}Si_{diatom}$ can be interpreted to reflect changes in the relative rate of nutrient (silicic acid) utilisation and supply in the photic zone (Fig. S2).

An increase (decrease) in $\delta^{30}Si_{diatom}$ could be caused by: 1) an increase (decrease) in biogenic silicic acid utilisation due to the isotope fractionation associated with this process; 2) a decrease (increase) in nutrient supply to the photic zone, which replenishes the pool of nutrients and their isotope composition; or 3) a combination of these two processes. In calculating rates of photic zone silicic acid utilisation and supply (see Equations in Methods Section which are repeated below for clarity) a series of assumptions are required, each of which are examined in the sections below:

$$\text{Si(OH)}_{4(\text{utilisation})} = (1 - \frac{\delta^{30}\text{Si}_{\text{diatom}} - 1.71}{1.61}) \bullet 100 \qquad (5)$$

$$\text{Si(OH)}_{4(\text{supply})} = \frac{\text{BSi MAR}_{\text{sample}}/\text{BSi MAR}_{\text{2005CE}}}{\text{Si(OH)}_{4(\text{utilisation})}/\text{Si(OH)}_{4(\text{utilisation-2005CE})}} \qquad (6)$$

Dissolution

The use of ($\delta^{30}Si_{diatom}$) in palaeoenvironmental research requires that the isotopic composition of living diatoms in the photic zone is faithfully transported into diatoms buried in the sediment record following their deposition at the surface-sediment interface[52]. This issue is particularly important in Lake Baikal, with only c. 1% of diatoms in the photic zone ultimately preserved in the sediment record[53,54] and dissolution indices indicating that up to 60% of diatom frustules preserved over the last 1,000 years have undergone some form of dissolution[18,55]. However, measurements from sediment traps distributed down the entire depth of Lake Baikal's water column and at the surface sediment show no alteration in $\delta^{30}Si_{diatom}$, indicating that any dissolution during sinking or sedimentation in Lake Baikal does not modify $\delta^{30}Si_{diatom}$[48].

The high rate of diatom dissolution does potentially impact sediment biogenic silica concentrations and so calculation of $Si(OH)_4$ supply (equation 6). The $Si(OH)_4$ supply record presented in this study assumes that the degree of biogenic silica dissolution through the water column and within the sediment record has remained unchanged over the analysed interval. This assumption can be tested through the use of a diatom dissolution index[56]:

$$\text{DDI} = \frac{\sum_{i=1}^{n} x_{1i}}{\sum_{i=1}^{n} x_{1i} + \sum_{i=1}^{n} x_{2i}} \qquad (7)$$

in which n is the number of diatom frustules ($n \geq 300$), x_{1i} is the number of pristine diatom frustules and x_{2i} is the number of diatoms frustules showing any sign of dissolution. From this the

raw BSi mass accumulation rate (MAR) concentration record can be corrected for dissolution and used to re-calculate changes in silicic acid supply to the photic zone (equation 6):

$$\text{BSi MAR [dissolution corrected]} = \frac{\text{BSi (wt.\%as SiO}_2\text{)}}{\text{DDI}} \bullet \text{MAR} \qquad (8)$$

The results from this show that whilst reconstructed rates of silicic acid supply are higher when the BSi data is corrected for dissolution, the overlying trend of a significant increase in nutrient (silicic acid) supply from the 20th Century onwards remain valid and is in agreement with concordant increases in *Ulnaria acus*, a diatom often associated with high dissolved silica concentrations[57,58] (Fig. S3). In this study we elect not to use the dissolution corrected nutrient supply record due to the uncertainties associated with individual indices[59,60]. We also avoid using diatom biovolume accumulation rates in place of BSi MAR since, although relatively well established for Lake Baikal[61], there remains considerable uncertainty in the calculation of species biovolume coefficients and over their potential to vary with time and changing environmental conditions, including changes in nutrient availability.

$\delta^{30}Si(OH)_4$

Although nutrients are primarily supplied to the lake through riverine inputs[33,34,62], intra- and inter-annual nutrient cycling in Lake Baikal is regulated by vertical mixing in the water column, which transports regenerated nutrients from deep waters in Lake Baikal into the photic zone[33,34]. Due to this cycling and the volume of water in Lake Baikal, the isotopic and DSi signature of riverine waters entering the lake is minimal beyond the immediate area around a river mouth[37]. As such, the $\delta^{30}Si$ composition of silicic acid ($\delta^{30}Si(OH)_4$) supplied to the photic zone and utilised by diatoms is best described by the $\delta^{30}Si$ composition of deep water ($\delta^{30}Si_{lake}$), constrained at 1.71‰ (1σ = 0.10‰)[37]. Whilst it is not possible to account for how $\delta^{30}Si_{lake}$ may have varied over time, the residence time of $Si(OH)_4$ in the south basin of Lake Baikal is 100–170 years[43,44], minimising the risk that large changes have occurred over the studied time interval.

Core chronology

All ^{14}C dating was completed at the Gliwice Radiocarbon Laboratory, Silesian University of Technology. The total organic carbon was prepared from the sediment by treatment with 0.5M HCl, subsequent rinse with DI water and drying. The samples were combusted in VarioMicroCube elemental analyser (Elementar™) coupled to AGE-3 graphitisation equipment[63]. ^{14}C concentration was determined in DirectAMS laboratory, Bothell, WA, USA[64] (Table S1).

New ^{14}C and existing ^{210}Pb dates[65] were combined using the Bacon software[66] to establish an age-depth model as well as an age interval for each analysed sample (Fig. S4). The curve IntCal13 was used for calibration of ^{14}C results[67]. For calculations, the cores were divided into 0.5-cm-thick sections. The priors used were accumulation rate 200 yr/cm of gamma distribution with shape 1.5. The default memory prior was used, which is a beta distribution with the parameters mem.strength = 4 and mem.mean = 0.7. The models were extrapolated to match the range of proxy data and the ages and uncertainties for required depths generated. Throughout the paper, the weighted mean age was used as an approximate age, along with 1σ uncertainty.

^{14}C chronologies are widely used in Lake Baikal, due to their unrivalled ability to date Holocene and late glacial sediments[68-70]. Some uncertainties exist over ^{14}C dating in Lake Baikal, with evidence for non-zero surface sediment ages, different sources of organic carbon (old carbon effect) and ^{14}C reservoir effects[71,72]. These artefacts do not impact the findings of this current study, with 20th and 21st Century increases in nutrient utilisation and Ekman transport inferred from sections of the age-model based on ^{210}Pb dating, the robustness of which has been demonstrated in Lake Baikal[73].

Ekman transport

Due to Lake Baikal's depth (maximum depth of 1,642 m) and climatic conditions, a key contribution to vertical mixing is given by periodic deep mixing events controlled by thermobaricity, i.e. the combined dependence of water density on temperature and pressure. According to this physical property of water, wind-driven inshore Ekman transport can trigger thermobarically unstable conditions, generating deep coastal downwellings that can reach down to the bottom of Lake Baikal. Deep mixing events typically occur in December/January, before ice formation on the lake, and in May-June after ice-out in late spring when the lake is weakly inversely stratified[41,42,74-79]. Further deep water renewal events have been detected under-ice, although it remains unclear to what extent these were driven by Ekman transport before the onset of ice-cover on the lake[80]. Deep mixing events promote the vertical redistribution of dissolved substances along the water column through the transfer of large volumes of water (ranging between 10 and 100 km^3 per year[77,78,81,82]) from the surface to the bottom of the lake and vice-versa. Such vertical transport and mixing activity in the lake promotes oxygen replenishment in deep water (i.e., deep ventilation) and the reciprocal upwelling of nutrient-rich deep waters to the photic zone, which fuels primary productivity within the lake.

CERA-20C reanalysis data[83] (resolution = 125 km) around the barycentre of Lake Baikal (53.375^{o}N, 108.125^{o}E) shows a progressive strengthening of surface wind (height = 10 m) from 1901-2010 CE during months in which deep ventilation occurs in the lake (Fig. S5). This trend is consistent with a progressively increased probability of more intensive deep ventilation events and so deep water nutrient supply to Lake Baikal's photic zone during the 20th and 21st Century and follows previous analyses that showed deep ventilation in Lake Baikal is particularly sensitive to climate change through changes in wind forcing[79]. Whilst the horizontal resolution of the CERA-20C data is relatively coarse (125 km), ERA5 reanalysis data[84] from 1979 CE, with a finer horizontal resolution (30 km), averaged over the same area covered by the CERA-20C data, accurately captures wind seasonality over Lake Baikal (Fig. S6), confirms the wind patterns and the inter-annual variability and trends described by CERA-20C over the same interval (Fig. S5), thereby providing validation of the coarser reanalysis product.

Equations for calculating Ekman transport in the lake are reported in the methods section of the main manuscript, with anomalies for a given downwelling season (*s*) of year (*i*) calculated relative to the mean Ekman transport from 1990-2000 CE (Fig. S7). By reasonably assuming that all variables in Equations 4 and 5 in the Methods Section of the main text are constant, except for wind speed parallel to Lake Baikal's coast (*W*), Ekman transport (*M*) anomalies can be calculated as the anomalies of the square of directional wind (*W*) over Lake Baikal around the barycentre of the lake:

$$Anomaly\ \overline{M_c}(i,s) = \frac{\overline{M_c}(i,s)}{\overline{M_c}(1990-2000,s)} = Anomaly\ \overline{W_c^2}(i,s) = \frac{\overline{W_c^2}(i,s)}{\overline{W_c^2}(1990-2000,s)} \qquad (9)$$

where the subscript *c* indicates the cumulative calculated across periods when the wind both blows from the north-east and is interrupted by winds from other directions for less than 1 day. The overbar indicates averaging over each downwelling season (*s*) of a given year (*i*), to obtain a quantitative measure of the potential intensity of deep ventilation in that season and year.

Diatom assemblage data

Changes in Ekman transport nutrient supply to the photic zone, from the late 19th Century onwards, coincide with changes in the diatom record (Fig. S8 a-e). In addition to reductions in the ratio of autumn/spring blooming taxa, significant declines also occur through the 20th and 21st Century in the wider diatom community (PCA axis 1) and composition turnover - the change in species composition and relative abundances - (DCCA axis 1)[85].

Changes in composition turnover, beginning in the late 19th Century and pre-dating other anthropogenic impacts on the lake, are strongly associated with reduced relative abundances for

the endemic *Crateriportula inconspicua* ($r = +0.75$; $p < 0.005$), (Fig. S8f). Whilst the ecology of *Cyclotella inconspicua* is poorly understood, phytoplankton monitoring has found *C. inconspicua* cells to be most abundant during autumn overturn[53]. However, *Cyclotella minuta* is the only pelagic diatom in Lake Baikal with a notable autumnal bloom, attributed to its ability to survive warm summer surface water temperatures (SWT) that result in a competitive advantage at the end of summer stratification[86]. Although *C. minuta* is only weakly associated with declining DCCA score (0.141; $p = 0.511$), together *C. inconspicua* and *C. minuta* suggest a significant decline in autumnal productivity from the 19th Century (Fig. S8 f-g).

In addition to the autumnal decline in *C. inconspicua* and, *C. minuta*, the increase in diatom composition turnover is strongly associated with increases in the endemic *Aulacoseira skvortzowii* ($r = -0.68$, $p < 0.005$) and cosmopolitan *Ulnaria acus* ($r = -0.83$; $p < 0.005$) (Fig. S8 h-i). *A. skvortzowii* is adapted to cold water conditions[87] whilst *U. acus* is a thermally tolerant taxon associated with higher dissolved silica concentrations in the water column[58,88]. Whilst previous work has attributed recent community changes in Lake Baikal's diatom community to warmer SWT and enhanced summer stratification (Fig. S8j)[55,89,] the concordant change between nutrient supply and diatom community changes from the late 19th Century, including increases in *U. acus*, suggests that community shifts over the last 150 years are a function of both nutrient supply and SWT. *A. skvortzowii* and *U. acus* also both require strong winds/currents in Lake Baikal for their cells to be transported from littoral to pelagic waters. The correlated increases in wind speed, Ekman transport driven nutrient supply, *A. skvortzowii* and *U. acus* therefore lends further support to our suggestion that increased photic zone nutrient supply has significantly impacted recent diatom changes in Lake Baikal (Fig. S8h-j), by accounting for 23.4% ($p = 0.002$) of community variation. All ordinations were calculated using CANOCO5[90].

The other dominant taxon in Lake Baikal, the endemic *Aulacoseira baicalensis*, exhibits a weak curvilinear trend through the 20th-21st Century (Fig. 8k). Whilst *A. baicalensis* needs relatively high concentrations of both silicic acid and turbulence in the photic zone to grow, the taxa is also adapted to cold water, low light conditions[91]. Although speculative, the increase in *A. baicalensis* up to c. 1950 CE may be linked to increasing photic zone nutrient supply, with the more recent (post-1950 CE) decline linked to increasing SWT, causing its heavily silicified cells to sink deeper into the water column (Fig. S8j-k).

Generalized additive model

Generalized additive models (GAM) were calculated with restricted maximum likelihood (REML) smoothness selection using the *mgcv* package in R[50,92,93]. To account for temporal autocorrelation, all models included a continuous-time first-order autoregressive (CAR(1)) process[94]. Intervals of significant temporal change in a GAM were detected using the first order derivative of the fitted trend and a 95% simultaneous confidence interval using the *gratia* package in R[50,94,95]. Additional breakpoint analyses were carried out using the *segmented* package in R[50,96].

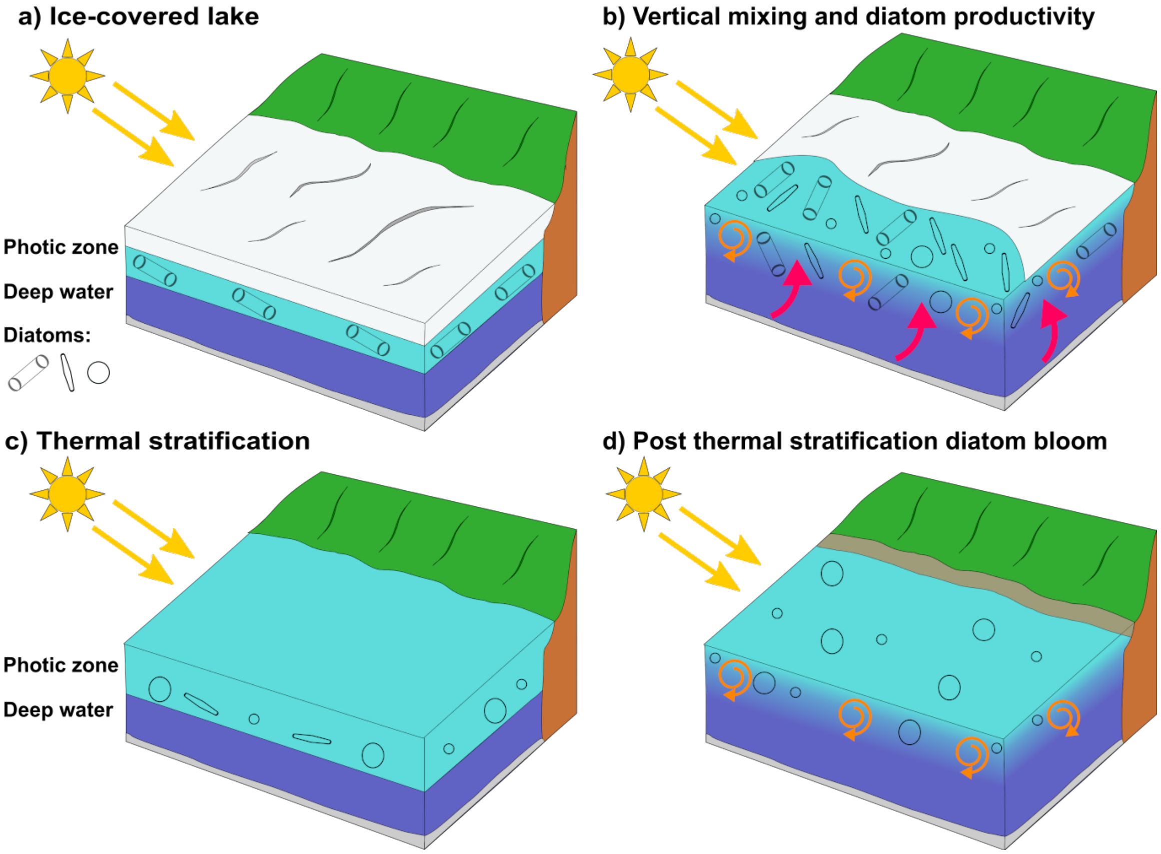

Fig. S1. Dominant seasonal controls on diatom productivity in Lake Baikal a, Ice/snow cover over the lake from January to May inhibits diatom blooms, although important under-ice growth occurs, with an increase during ice break-up. b, Ice break-up from May onwards allows turbulent mixing within the uppermost sections of the water column. Combined with coastal downwelling (typical months = May-June and December-January), and the associated upwelling and build-up of nutrient-rich deep waters in the photic zone, large diatom blooms occur. c, Thermal stratification of the lake in summer reduces nutrient availability for diatoms, with productivity instead dominated by autotrophic pico-cyanobacteria. d, Breakdown of the stratification in autumn months restarts the turbulent mixing of nutrients into the photic zone. Due to the absence of coastal downwelling and deep water supply of nutrients to the photic zone, the autumnal bloom is typically smaller than the spring bloom.

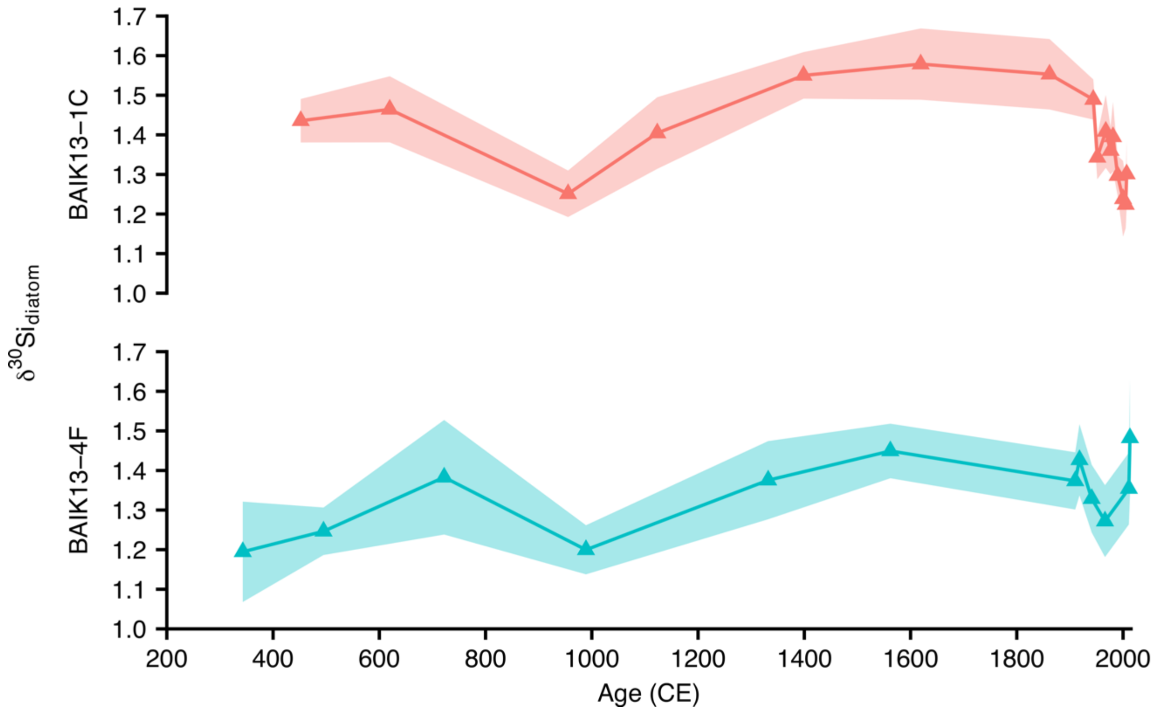

Fig. S2. $\delta^{30}Si_{diatom}$ in cores BAIK13-1C and BAIK13-4F in Lake Baikal (see Fig. 1 in the main text for core location). Given the proximity of the two sites and their well constrained age-models, these records are combined to create a composite record which are then used to reconstruct changes in photic zone silicic acid utilisation/supply (see main text). Shaded polygons reflect the 1σ uncertainty derived from Monte Carlo simulations (10,000 replicates).

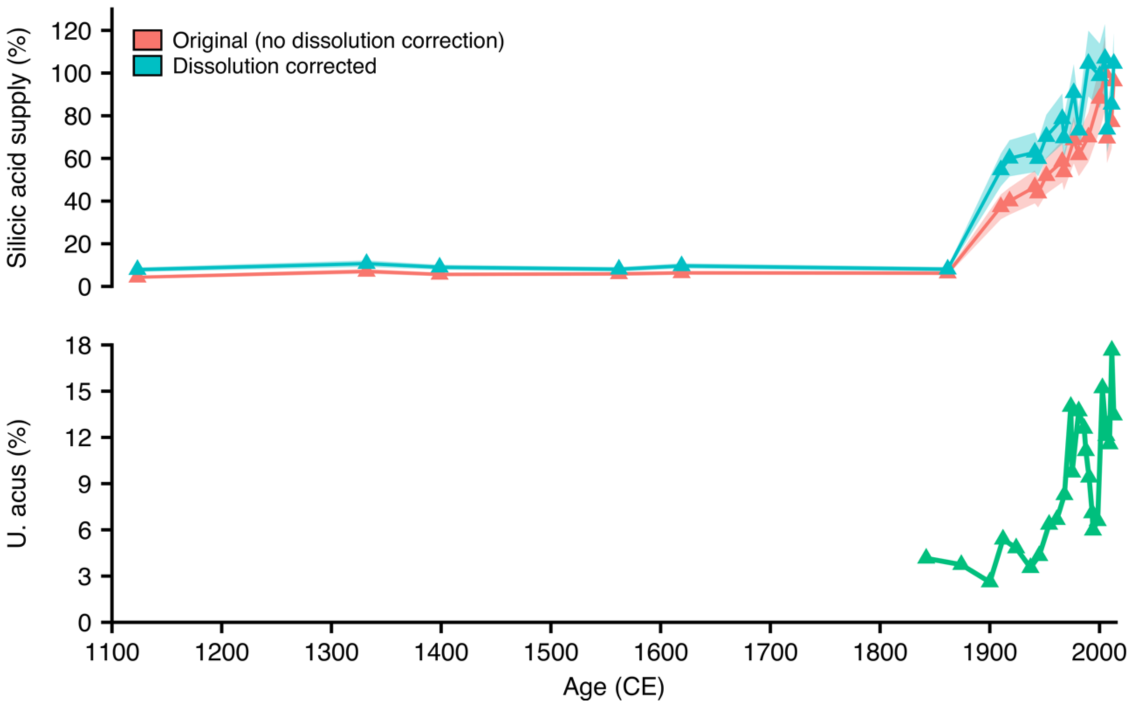

Fig. S3. Changes in photic zone silicic acid supply (relative to a value of 100% at 2005 CE). The original supply record used in the manuscript contains no correction for diatom dissolution. The dissolution corrected supply record uses diatom dissolution indices[18,55] to correct the BSi MAR record for differential levels of diatom dissolution through the sediment core. Shaded regions reflect the 1σ uncertainty derived from Monte Carlo simulations (10,000 replicates). Higher rates of silicic acid supply in the 20th and 21st Century are in agreement with relative abundance increases in the diatom *U. acus* at core site BAIK13-7[18,55] (Fig. 1).

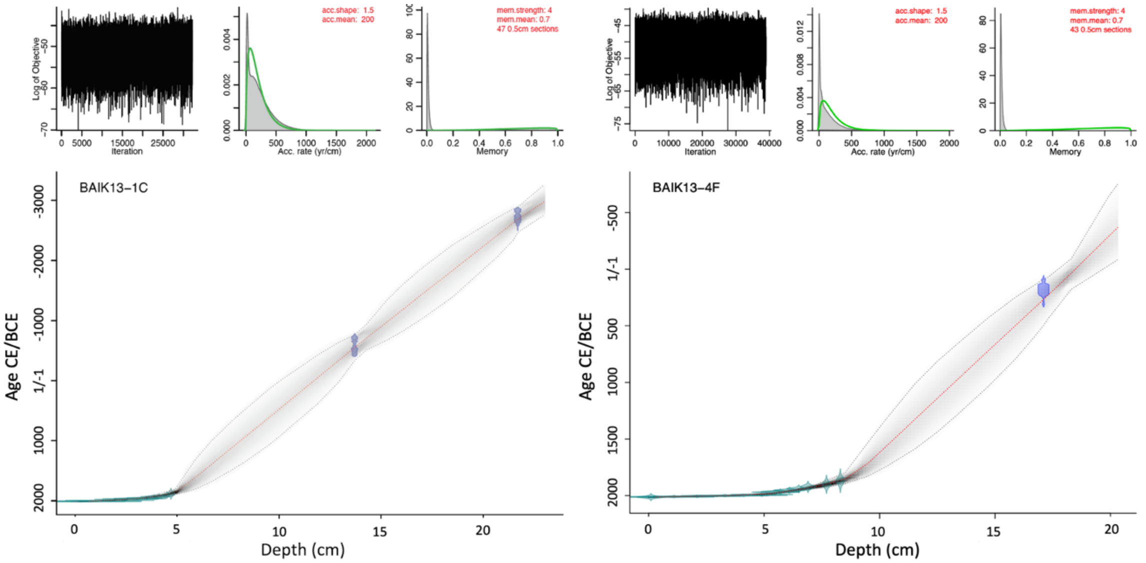

Fig. S4. Age-depth models for BAIK13-1C and BAIK13-4F. Models were generated with the use of Bacon[66]. Upper panels: MCMC iterations structure on the left; prior (green) and posterior (grey) distributions for accumulation rate in the middle; prior and posterior distributions for memory on the right. Lower panels: red dotted curve shows the age-depth model, the grey-shaded area presents 95% probability intervals; ^{210}Pb ages are represented by Gaussian distributions in green and ^{14}C calibrated ages are shown as blue distributions.

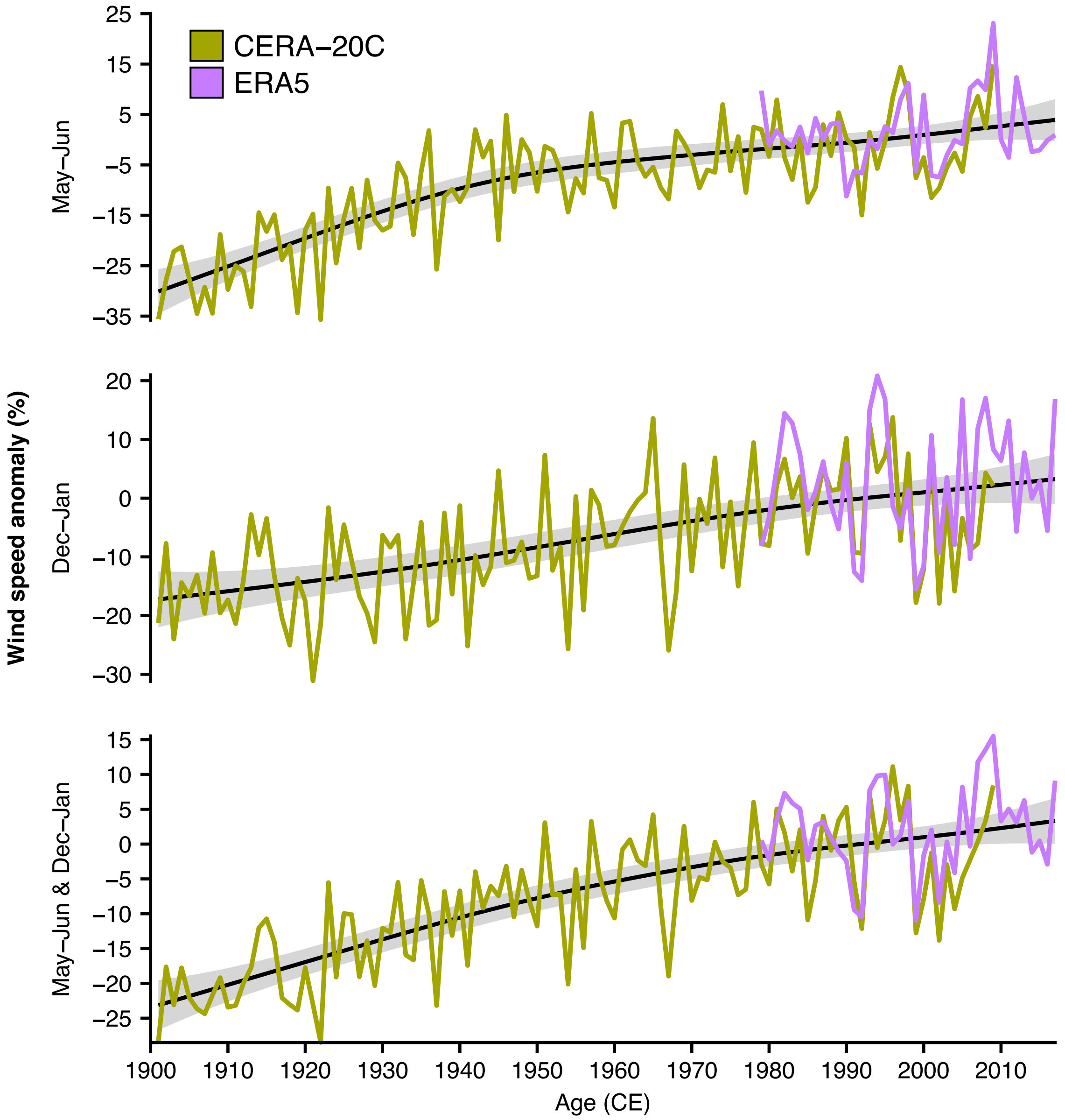

Fig. S5. CERA-20C[83] and ERA5[84] wind speed reanalysis data from the barycentre of Lake Baikal during the typical periods of coastal downwelling (May-June and December-January). All data are anomalies relative to a baseline period of 1990-2000 CE. Black line and grey confidence interval show a generalized additive model fitted to each interval using R[50].

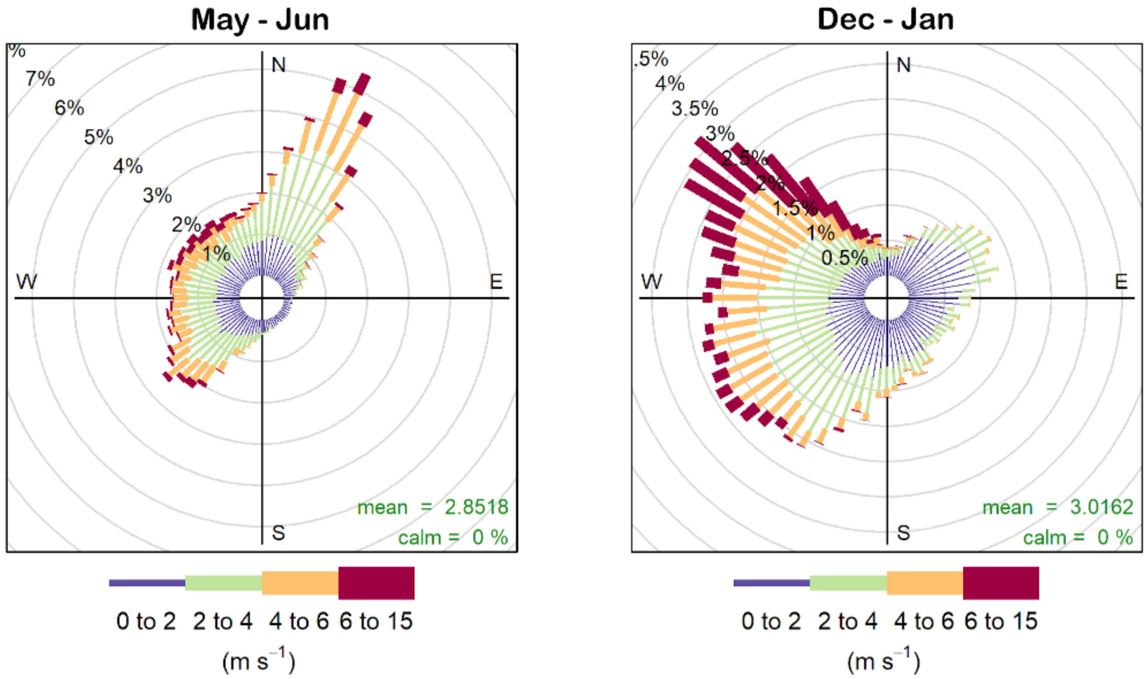

Fig. S6. ERA5 wind analyses frequency counts by wind direction from 1979-2019 CE. The wind roses are consistent with the typical wind fields observed at the lake[97] and with those available from downscaled regional atmospheric models for the region (see Tsimitri et al., 2015[80] for the seasonal climatology of wind in the south basin of Lake Baikal).

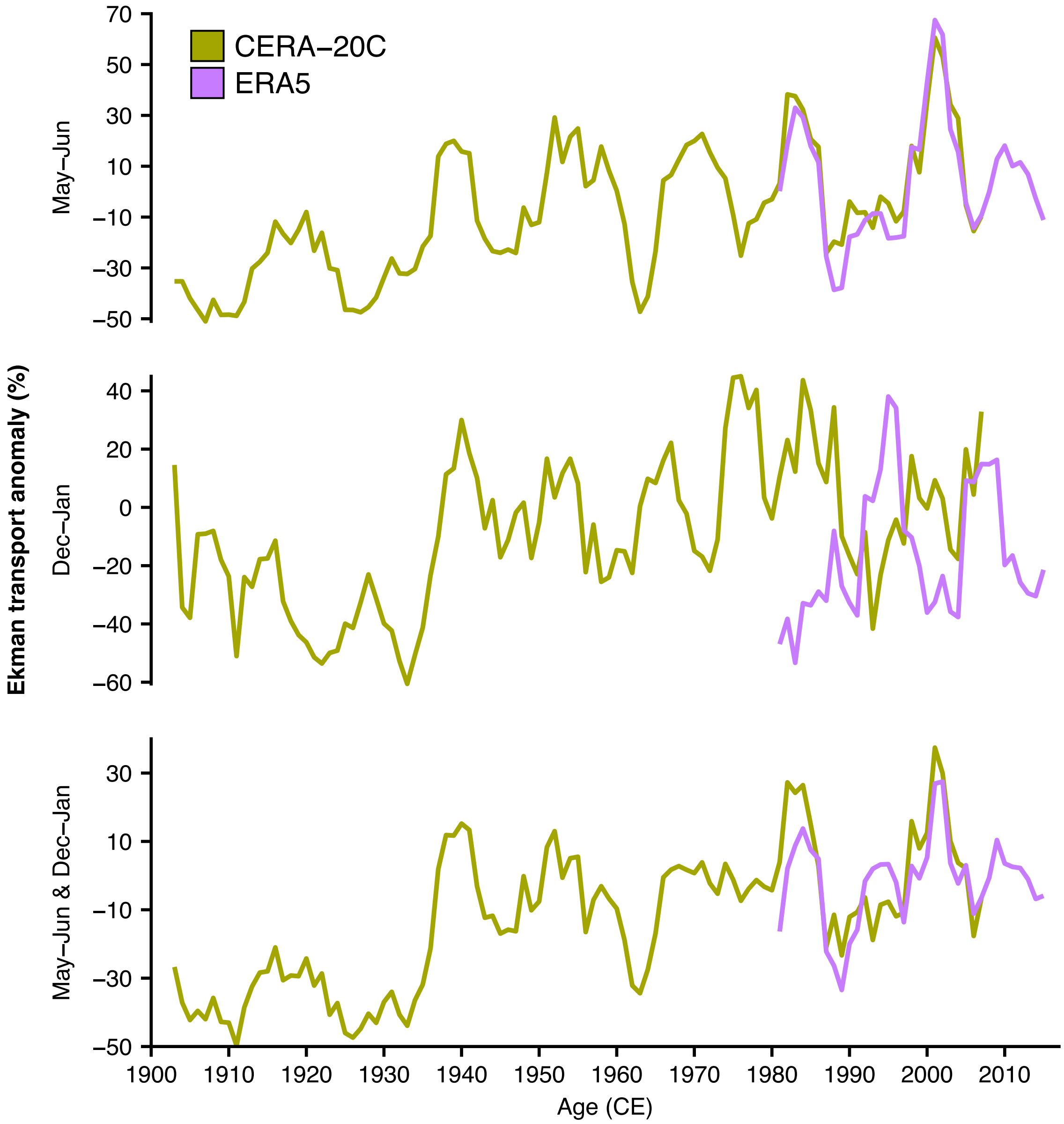

Fig. S7. Modelled Ekman transport in Lake Baikal with a 5-year running mean during the typical periods of downwelling (May-June and December-January). All anomalies are relative to a baseline period of 1990-2000 CE with wind data which drives Ekman transport taken from CERA-20C and ERA5 reanalysis data from the barycentre of Lake Baikal.

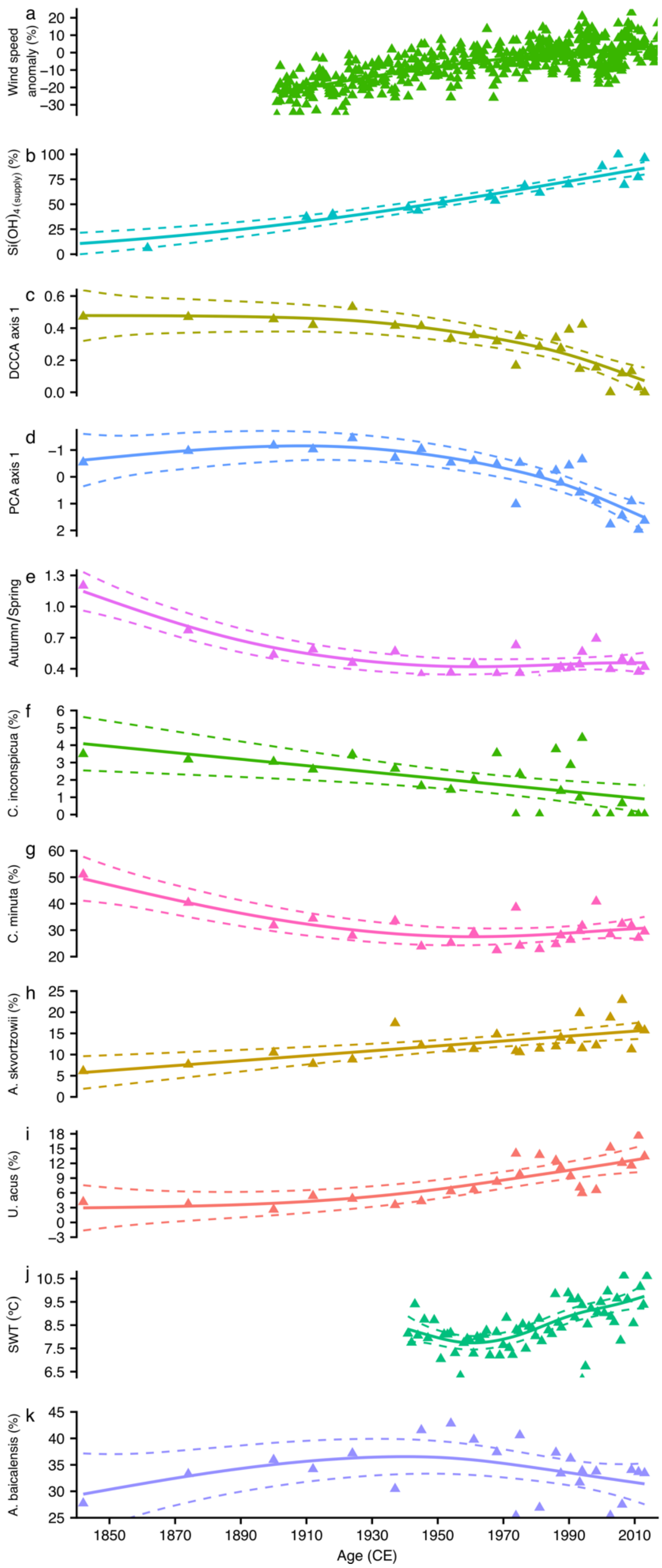

a
Wind speed anomaly (%)
20
10
0
−10
−20
−30
b
Si(OH)4 (supply) (%)
100
75
50
25
0
c
DCCA axis 1
0.6
0.4
0.2
0.0
d
PCA axis 1
−1
0
1
2
e
Autumn/Spring
1.3
1.0
0.7
0.4
f
C. inconspicua (%)
6
5
4
3
2
1
0
g
C. minuta (%)
60
50
40
30
20
h
A. skvortzowii (%)
25
20
15
10
5
0
i
U. acus (%)
18
15
12
9
6
3
0
−3
j
SWT (°C)
10.5
9.5
8.5
7.5
6.5
k
A. baicalensis (%)
45
40
35
30
25
1850
1870
1890
1910
1930
1950
1970
1990
2010
Age (CE)

Fig. S8. Diatom community changes in Lake Baikal at core site BAIK13-7[18,55] (Fig. 1). a, CERA-20C[83] and ERA5[84] wind speed reanalysis anomaly data, relative to a baseline period of 1990-2000 CE, from the barycentre of Lake Baikal during the typical periods of downwelling (May-June and December-January). b, Changes in photic zone silicic acid supply (relative to a value of 100% at 2005 CE). c, Detrended canonical correspondence analysis (DCCA) axis 1 scores reflecting diatom composition turnover. d, Principal components analysis (PCA) axis 1 scores reflect changing diatom taxa composition. e, Ratio of autumn/spring taxa. f, Relative abundance of *C. inconspicua*. g, Relative abundance of *C. minuta*. h, Relative abundance of *A. skvortzowii*. i, relative abundance of *U. acus*. j, Mean lake surface water temperatures (SWT) (May-October) from shoreline locations across Lake Baikal[15]. k, Relative abundance of *A. baicalensis*. For all panels, trend lines (solid) and confidence intervals (dashed lines) show a generalized additive model fitted to each time series.

Table S1. New ^{14}C dates used in this study.

Core	Depth (cm)	^{14}C Age (BP)	Calibrated Age (CE/BCE)	Modelled age (CE/BCE) (weighted mean ± 1 σ)
BAIK13-1C	13.7	2445 ± 30	68.2% probability<br>735-670 BCE (20.1%)<br>660-650 BCE (6.2%)<br>545-430 BCE (41.8%)<br>95.4% probability<br>750-685 BCE (25.1%)<br>670-615 BCE (13.3%)<br>590-410 BCE (57.0%)	550 ± 110 BCE
BAIK13-1C	21.7	4135 ± 30	68.2% probability<br>28600 (13.6%) 2835 BCE<br>2820-2810 BCE (5.5%)<br>2755-2720 BCE (17.3%)<br>2705-2635 BCE (31.8%)<br>95.4% probability<br>2870-2620 BCE (95.4%)	2690 ± 110BCE
BAIK13-4F	17.1	1835 ± 30	68.2% probability<br>130-215 CE (68.2%)<br>95.4% probability<br>85-250 CE (95.4%)	275 ± 105CE

Dataset S1 (separate file). $\delta^{30}Si_{diatom}$ and absolute analytical uncertainty (2σ) together with reconstructed rates of silicic acid utilisation and supply to the photic zone with the 1σ uncertainty derived from Monte Carlo simulations (10,000 replicates).

Dataset S2 (separate file). Biogenic silica concentrations from core BAIK13-1C.

Dataset S3 (separate file). CERA-20C wind speed reanalysis data from the barycentre of Lake Baikal during the typical periods of coastal downwelling (May-June and December-January). All data are anomalies relative to a baseline period of 1990-2000 CE.

Dataset S4 (separate file). Modelled Ekman transport anomalies during the typical periods of coastal downwelling (May-June and December-January). All anomalies are relative to a baseline period of 1990-2000 CE.

SI References

1. R.R. Seal, W.C. Shanks, Oxygen and hydrogen isotope systematics of Lake Baikal, Siberia: implications for palaeoclimate studies. Limnol. Oceanogr. 43, 1251-1261 (1998).
2. O.A. Timoshkin. “Biodiversity of Baikal fauna: state-of-the-art (Preliminary analysis)” in New Scope on the Boreal Ecosystems in East Siberia, E. Wada, O.A. Timoshkin, N. Fujita, K. Tanida, Eds. Proceedings of the International Symposium, Kyoto, Nov. 23–25 1994, (DIWPA Ser. No.2). The Siberian Branch of the Russian Academy of Sciences Press, Novosibirsk, pp. 35–76 (1997).
3. G. Fryer, Comparative aspects of adaptive radiation and speciation in Lake Baikal and the great rift lakes of Africa. Hydrobiologia 211, 137-146 (1991).
4. UNESCO, Convention concerning the protection of the world cultural and natural heritage. World Heritage Committee, Merida, Mexico. https://whc.unesco.org/archive/repcom96.htm (1996).
5. L.S. Kravtsova, L.A. Izhboldina, I.V. Khanaev, G.V. Pomazkina, E.V. Rodionova, V.M. Domysheva, M.V. Sakirko, I.V. Tomberg, T,Ya. Kostornova, O.S. Kravchenko, A.B. Kupchinsky, Nearshore benthic blooms of filamentous green algae in Lake Baikal. J. Great Lakes Res. 40, 441-448 (2014).
6. O.A. Timoshkin, D.P. Samsonov, M. Yamamuro, M.V. Moore, O.I. Belykh, V.V. Malnik, , M.V. Sakirko, A.A. Shirokaya, N.A. Bondarenko, V.M. Domysheva, G.A. Fedorova, A.I. Kochetkov, A.V. Kuzmin, A.G Lukhnev., O.V. Medvezhonkova, A.V. Nepokrytykh, E.M. Pasynkova, A.E. Poberezhnaya, N.V. Potapskaya, N.A. Rozhkova, N.G. Sheveleva, I.V. Tikhonova, E.M. Timoshkina, I.V. Tomberg, E.A. Volkova, E.P. Zaitseva, Yu.M. Zvereva, A.B. Kupchinsky, N.A. Bukshuk, Rapid ecological change in the coastal zone of Lake Baikal (East Siberia): Is the site of the world's greatest freshwater biodiversity in danger? J. Great Lakes Res. 42, 487-497 (2016).
7. T.V. Khodzher, V.M. Domysheva, L.M. Sorokovikova, M.V. Sakirko, I.V. Tomberg, Current chemical composition of Lake Baikal water. Inland Waters 7, 250-258 (2007).
8. O.A. Timoshkin, Coastal zone of the world’s great lakes as a target field for interdisciplinary research and ecosystem monitoring: Lake Baikal (East Siberia). Limnology and Freshwater Biology 81-97 (2018).
9. S. Roberts, J.K. Adams, A.W. Mackay, G.E.A. Swann, S. McGowan, N.L. Rose, V. Panizzo, H. Yang, E. Vologina, M. Sturm, A.A. Shchetnikov, Mercury loading within the Selenga River basin and Lake Baikal, Siberia. Environ. Pollut. 259, 113814 (2020).
10. M.N. Shimaraev, L.N. Kuimova, V.N. Sinyukovich, V.V. Tsekhanovskii, Manifestation of Global Climatic Changes in Lake Baikal during the 20th Century. Dokl. Earth Sci. 383, 288-291 (2002).
11. M.N. Shimaraev, V.N. Sinyukovich, L.N. Sizova, E.S. Troitskaya, Heat balance of Lake Baikal and the relationship of its ice-thermal and water regime with global atmospheric circulation in the Northern Hemisphere during the modern period. Limnology and Freshwater Biology 1, 10-14 (2018).

12. M.N. Shimaraev, L.N. Sizova, E.S. Troitskaya, L.N. Kuimova, N.I. Yakimova, Ice-thermal Regime of Lake Baikal under Conditions of Modern Warming (1950-2017). Russ. Meteorol. Hydrol. 44, 679-686 (2019).
13. M.C. Todd, A.W. Mackay, Large scale climatic controls on Lake Baikal ice cover. J. Climatol. 16, 3186-3199 (2003).
14. S.E. Hampton, L.R. Izmest'eva, M.V. Moore, S.L. Katz, B. Dennis, E.A. Silow, Sixty years of environmental change in the world's largest freshwater lake - Lake Baikal, Siberia. Glob. Chang. Biol. 14, 1947-1958 (2008).
15. T.G. Potemkina, V.L. Potemkin, A.L. Fedotov, Climatic factors as risks of recent ecological changes in the shallow zone of Lake Baikal. Russ. Geol. Geophys. 59, 556–565 (2018).
16. S.E. Hampton, D.K. Gray, L.R. Izmest'eva, M.V. Moore, T. Ozersky, The Rise and Fall of Plankton: Long-Term Changes in the Vertical Distribution of Algae and Grazers in Lake Baikal, Siberia. PLoS ONE 9, e88920 (2014).
17. L.R. Izmest'eva, M.V. Moore, S.E. Hampton, C.J. Ferwerda, D.K. Gray, K.H. Woo, H.V. Pislegina, L.S. Krashchuk, S.V. Shimaraeva, E.A. Silow, Lake-wide physical and biological trends associated with warming in Lake Baikal. J. Great Lakes Res. 42, 6-17 (2016).
18. S.L. Roberts, G.E.A. Swann, S. McGowan, V.N. Panizzo, E.G. Vologina, M. Sturm, A.W. Mackay, Diatom evidence of 20th century ecosystem change in Lake Baikal, Siberia. PLoS ONE 13, e0208765 (2018).
19. N.A. Bondarenko, T. Ozersky, L.A. Obolkina, C. Tikhonova, E.G. Sorokovikova, M.V. Sakirko, S.A. Potapov, V.V. Blinov, A.A. Zhdanov, O.I. Belykh, Recent changes in the spring microplankton of Lake Baikal, Russia. Limnologica 75, 19-29 (2019).
20. M.V. Moore, S.E. Hampton, L.R. Izmest'eva, E.A. Silow, E.V. Peshkova, B.K. Pavlov, Climate change and the World's "Sacred Sea"-Lake Baikal, Siberia. Bioscience 49, 405-417 (2009).
21. M.V. Moore, Trophic coupling of the microbial and the classical food web in Lake Baikal, Siberia. Freshwater Biol. 64, 138-151 (2019).
22. S. Fietz, A. Nicklisch, An HPLC analysis of the summer phytoplankton assemblage in Lake Baikal. Freshwater Biol. 49, 332-345 (2004).
23. S. Fietz, G. Kobanova, L. Izmesteva, A. Nicklisch, Regional, vertical and seasonal distribution of phytoplankton and photosynthetic pigments in Lake Baikal. J. Plankton. Res. 27, 793-810 (2005).
24. G.I. Popovskaya, Ye.U. Likhoshway, S.I. Genkal, A.D. Firsova, The role of endemic diatom algae in the phytoplankton of Lake Baikal. Hydrobiologia 568, 87-94 (2006).
25. G.I. Popovskaya, M.V. Usol'tseva, V.M. Domysheva, M.V. Sakirko, V.V. Blinov, T.V. Khodzher, The spring phytoplankton in the pelagic zone of Lake Baikal during 2007-2011. Geogr. Nat. Resour. 36, 253-262 (2015).
26. V.N. Panizzo, S. Roberts, G.E.A. Swann, S. McGowan, A.W. Mackay, E. Vologina, V.H. Pashley, M.S.A. Horstwood, Spatial differences in dissolved silicon utilization in Lake Baikal, Siberia: Examining the impact of high diatom biomass events and eutrophication. Limnol. Oceanogr. 63, 1562-1578 (2018).
27. B.W. Skvortzow, Diatoms from Lake Baikal. Philippine Journal of Science 62, 293-377 (1937).
28. R.J. Flower, G. Pomazkina, E. Radionova, D.M. Williams, "Local and meso-scale diversity patterns of benthic diatoms in Lake Baikal" in Proceedings of the 17th Diatom Symposium, M. Poulin, Ed. (Biopress Ltd, 2002), pp. 69-92.
29. R.J. Flower, A review of diversification trends in diatom research with special reference to taxonomy and environmental applications using examples from Lake Baikal and elsewhere. Proceedings of the Californian Academy of Sciences, 56, 107-128 (2005).
30. L.Z. Granina, E.B. Karabanov, M.K. Shimaraeva, D.F. Williams, V.M. Kuptsov, "Biogenic silica of Baikal bottom sediments used for palaeo reconstruction" in International project on palaeolimnology and lake Cenozoic climate newsletter, vol. 6, (Universitätsverlag Wagner, 1992) pp. 53-59.

31. D.F. Williams, M.I. Kuzmin, A.A. Prokopenko, E.B. Karabanov, G.K. Khursevich, E.V. Bezrukova, The Lake Baikal drilling project in the context of a global lake drilling initiative. Quat. Int. 80/81, 3-18 (2001).
32. A.W. Mackay, The paleoclimatology of Lake Baikal: A diatom synthesis and prospectus. Earth-Sci. Rev. 82, 181-215 (2007).
33. E. Callender, L. Granina, Geochemical mass balance of major elements in Lake Baikal, Limnol Oceanogr. 42, 148-155 (1997).
34. B. Müller, M. Maerki, M. Schmid, E.G. Vologina, B. Wehrli, A. Wuest, M. Sturm, Internal carbon and nutrient cycling in Lake Baikal: sedimentation, upwelling, and early diagenesis. Global Planet. Change. 46, 101-124 (2005).
35. G.I. Popovskaya, Ecological monitoring of phytoplankton in Lake Baikal. Aquat. Ecosyst. Health. 3, 215-225 (2000).
36. D.H. Jewson, N.G. Granin, A.A. Zhdanov, R.Y. Gnatovsky, Effect of snow depth on under-ice irradiance and growth of *Aulacoseira baicalensis* in Lake Baikal. Aqua. Ecol. 43, 673-679 (2009).
37. V.N. Panizzo, G.E.A. Swann, A.W. Mackay, E. Vologina, L. Alleman, L. Andre, V.H. Pashley, M.S.A. Horstwood, Constraining modern day silicon cycling in Lake Baikal. Global Biogeochem. Cy. 31, 556-574 (2017).
38. O.I. Belykh, G. Ekaterina, T. Sorokovikova, A. SaphonovaI. V. Tikhonova, Autotrophic picoplankton of Lake Baikal: Composition, abundance and structure. Hydrobiologia 568, 9-17 (2006).
39. Y. Satoh, T. Katano, T, Satoh, O. Mitamura, K. Anbutsu, S. Nakano, H. Ueno, M. Kihira, V. Drucker, Y. Tanaka, T. Mimura, Y. Watanabe, M. Sugiyama, Nutrient limitation of the primary production of phytoplankton in Lake Baikal. Limnology 7, 225-229 (2006).
40. D.R. O'Donnell, P. Wilburn, E.A. Silow, L.Y. Yampolsky, E. Litchman, Nitrogen and phosphorus colimitation of phytoplankton in Lake Baikal: Insights from a spatial survey and nutrient enrichment experiments. Limnol. Oceanogr. 62, 1383-1392 (2017).
41. R.F. Weiss, E.C. Carmak, V.M. Koropalov, Deep-water renewal and biological production in Lake Baikal. Nature 349, 665-669 (1991).
42. P.D. Killworth, E.C. Carmack, R.F. Weiss, R. Matear, Modeling deep-water renewal in Lake Baikal, Limnol. Oceanogr. 41, 1521-1538 (1996).
43. K.K. Falkner, M. Church, C.I. Measures, G. LeBaron, D. Thouron, C. Jeandel, M.C. Stordal, G.A. Gill, R. Mortlock, P. Froelich, L.-H. Chan, Minor and trace element chemistry of Lake Baikal, its tributaries, and surrounding hot springs. Limnol. Oceanogr. 42, 329-345 (1997).
44. M.N. Shimaraev, V.M. Domysheva, Climate and long-term silica dynamics in Lake Baikal. Russ. Geol. Geophys. 45, 310-316 (2004).
45. M.N. Shimaraev, V.M. Domysheva, Dynamics of dissolved silica in Lake Baikal, Dokl Earth Sci. 387, 1075-1078 (2002).
46. I.L. Barnes, L.J. Moore, L.A. Machlan, T.J. Murphy, W.R. Shields, Absolute isotopic abundance ratios and atomic weight of a reference sample of silicon. J. Res. Natl. Bur. Stand. 79A, 727-735 (1975).
47. J.N. Sutton, D.E. Varela, M.A. Brzezinski, C.P. Beucher, Species-dependent silicon isotope fractionation by marine diatoms. Geochem. Cosmochim. Ac. 104, 300-309 (2013).
48. V.N. Panizzo, G.E.A. Swann, A.W. Mackay, E. Vologina, M. Sturm, V. Pashley, M.S.A. Horstwood, Insights into the transfer of silicon isotopes into the sediment record. Biogeosciences 13, 147-157 (2016).
49. C.H. Leschinski, MonteCarlo: Automatic Parallelized Monte Carlo Simulations. R package version 1.0.6. https://CRAN.R-project.org/package=MonteCarlo (2019).
50. R Core Team, R: A language and environment for statistical computing. R Foundation for Statistical Computing, Vienna, Austria. https://www.R-project.org/ (2020).
51. C.L. de la Rocha, M.A. Brzezinski, M.J. DeNiro, Fractionation of silicon isotopes by marine diatoms during biogenic silica formation. Geochem. Cosmochim. Ac. 61, 5051-5056 (1997).

52. M.S. Demarest, M.A. Brzezinski, C.P. Beucher, Fractionation of silicon isotopes during biogenic silica dissolution. Geochem. Cosmochim. Ac. 73, 5572-5583 (2009).
53. D.B. Ryves, D.H. Jewson, M. Sturm, R.W. Battarbee, R.J. Flower, A.W. Mackay, N.G. Granin, Quantitative and qualitative relationships between planktonic diatom communities and diatom assemblages in sedimenting material and surface sediments in Lake Baikal, Siberia. Limnol. Oceanogr. 48, 1643-1661 (2003).
54. R.W. Battarbee, A.W. Mackay, D. Jewson, D.B. Ryves, M. Sturm, Differential dissolution of Lake Baikal diatoms: correction factors and implications for palaeoclimatic reconstruction. Global Planet. Change. 46, 75-86 (2005).
55. A.W. Mackay, R.J. Flower, A.E. Kuzmina, L.Z. Granina, N.L. Rose, P.G. Appleby, J.F. Boyle, R.W. Battarbee, Diatom succession trends in recent sediments from Lake Baikal and their relation to atmospheric pollution and to climate change. Philos. Trans. R. Soc. Lond. B 353, 1011-1055 (1998).
56. R.J. Flower, Y.V. Likhoshway, “Diatom preservation in Lake Baikal” in Diatom Algae as Indicators of the Changes of Climate and Environment: Fifth Workshop on Diatom Algae, M.A. Grachev, Ed. (Botanical Society Publications, 1993), pp. 77-78.
57. P. Kilham, S.S Kilham, Endless summer, internal loading processes dominate nutrient cycling in tropical lakes. Freshwater Biol. 23, 379-389 (1990).
58. J.P. Bradbury, Y.V. Bezrukova, G.P. Chernyaeva, S.M. Colman, G. Khursevich, J.W. King, Ye.V. Likoshway, A synthesis of post-glacial diatom records from Lake Baikal. J. Paleolimnol. 10, 213-252 (1994).
59. R.J. Flower, D.B. Ryves, Diatom preservation: differential preservation of sedimentary diatoms in two saline lakes. Acta Bot. Croat. 68, 381-399 (2009).
60. D.B. Ryves, R.W. Battarbee, S.C. Fritz, The dilemma of disappearing diatoms: Incorporating diatom dissolution data into palaeoenvironmental modelling and reconstruction. Quaternary Sci. Rev. 28, 120-136 (2009).
61. P. Rioual, A.W. Mackay, A diatom record of centennial resolution for the Kazantsevo Interglacial stage in Lake Baikal (Siberia). Global Planet. Change. 46, 199-219 (2005).
62. L. Granina, A chemical budget of Lake Baikal: a review. Limnol. Oceanogr. 42, 373-378 (1997).
63. L. Wacker, M. Nemec, J. Bourquin, A revolutionary graphitisation system: Fully automated compact and simple. Nucl. Instrum. Meth. B. 268, 931-934 (2010).
64. U. Zoppi, J. Crye, Q. Song, A. Arjomand, Performance evaluation of the new AMS system at Accium BioSciences. Radiocarbon 49, 173-182 (2007).
65. G.E.A. Swann, A.W. Mackay, E. Vologina, M.D. Jones, V.N. Panizzo, M.J. Leng, H.J. Sloane, A.M. Snelling, M. Sturm, Lake Baikal isotope records of Holocene Central Asian precipitation. Quaternary Sci. Rev. 189, 210–222 (2018).
66. M. Blaauw, J.A. Christen, Flexible paleoclimate age-depth models using an autoregressive gamma process. Bayesian Anal. 6, 457-474 (2011).
67. P.J. Reimer, E. Bard, A. Bayliss, J.W., Beck, P.G. Blackwell, C. Bronk Ramsey, P.M. Grootes, T.P. Guilderson, H. Haflidason, I. Hajdas, C. Hatte, T.J. Heaton, D.L. Hoffmann, A.G. Hogg, K.A. Hughen, K.F. Kaiser, B. Kromer, S.W. Manning, M. Niu, R.W. Reimer, D.A. Richards, E.M. Scott, J.R. Southon, R.A. Staff, C.S.M. Turney, J. van der Plicht, IntCal13 and Marine13 Radiocarbon Age Calibration Curves 0-50,000 Years cal BP. Radiocarbon 55, 1869-1887 (2013).
68. A.A. Prokopenko, D.F. Williams, E.B. Karabanov, G.K. Khursevich, Response of Lake Baikal ecosystem to climate forcing and pCO_2 change over the last glacial-interglacial transition. Earth Planet. Sc. Lett. 172, 239-253 (1999).
69. T. Watanabe, T. Nakamura, F.W. Nara, T. Kakegawa, M. Nishimura, M. Shimokawara, T. Matsunaka, R. Senda, T. Kawai, A new age model for the sediment cores from Academician ridge (Lake Baikal) based on high-time-resolution AMS ^{14}C data sets over the last 30 kyr: paleoclimatic and environmental implications. Earth Planet. Sc. Lett. 286, 347-354 (2009a).
70. A.W. Mackay, A.W.R. Seddon, M.J. Leng, G. Heumann, D.W. Morley, N. Piotrowska, P. Rioual, S. Roberts, G.E.A. Swann, Holocene carbon dynamics at the forest–steppe ecotone of southern Siberia. Global Change Biol, 23, 1942-1960 (2017).

71. S.M. Colman, G.A. Jones, M. Rubin, J.W. King, J.A. Peck, W.H. Orem, AMS radiocarbon analyses from Lake Baikal, Siberia: challenges of dating sediments from a large, oligotrophic lake. Quaternary Sci. Rev. 15, 669-684 (1996).
72. T. Watanabe, T. Nakamura, F. Nara, T. Kakegawa, K. Horiuchi, R. Senda, T. Oda, M. Nishimura, G.I. Matsumoto, T. Kawai, High time-resolution AMS ^{14}C data sets for Lake Baikal and Lake Hovsgol sediment cores: changes in radiocarbon age and linear sedimentation rates during the last glacial-Holocene period. Quatern. Int. 205, 12-20 (2009b).
73. P.G. Appleby, R.J. Flower, A.W. Mackay, N.L. Rose, Paleolimnological assessment of recent environmental change in Lake Baikal: sediment chronology. J. Paleolimnol. 20, 119-133 (1998).
74. M.N. Shimaraev, V.I. Verbolov, N.G. Granin, P.P. Sherstyankin, Physical limnology of Lake Baikal: a review, Irkutsk & Okayama: BICER (1994).
75. M.N. Shimaraev, E.S. Troitskaya, R.Yu. Gnatovskii, Modern Climate Changes and Deep Water Temperature of Lake Baikal. Dokl. Earth Sci. 427, 804-808 (2009).
76. M.N. Shimaraev, E.S. Troitskaya, V.V. Blinov, V.G. Ivanov, R.Yu. Gnatovskii, Upwellings in Lake Baikal. Dokl. Earth Sci. 442, 272-276 (2012).
77. A. Wüest, T.M. Ravens, N.G. Granin, O. Kocsis, M. Schurter, M. Sturm, Cold intrusions in Lake Baikal: Direct observational evidence for deep-water renewal, Limnol. Oceanogr. 50, 184-196 (2005).
78. M. Schmid, N.M. Budnev, N.G. Granin, M. Sturm, M. Schurter, A. Wüest, Lake Baikal deepwater renewal mystery solved. Geophys. Res. Lett. 35, 1-5 (2008).
79. S. Piccolroaz, M. Toffolon, The fate of Lake Baikal: how climate change may alter deep ventilation in the largest lake on Earth. Clim. Change 150, 181-194 (2018).
80. C. Tsimitri, B. Rockel, A. Wüest, N.M. Budnev, M. Sturm, M. Schmid, Drivers of deep-water renewal events observed over 13 years in the South Basin of Lake Baikal. J. Geophys. Res. Oceans 120, 1508-1526 (2015).
81. M.N. Shimaraev, R.Y. Gnatovskii, V.V. Blinov, V.G. Ivanov, Renewal of deep waters of Lake Baikal revisited. Dokl. Earth Sci. 438, 652-655 (2011).
82. S. Piccolroaz, M. Toffolon, Deep water renewal in Lake Baikal: A model for long-term analyses. J. Geophys. Res. Oceans 118, 6717-6733 (2013).
83. P. Laloyaux, E. de Boisseson, M. Balmaseda, J.-R. Bidlot, S. Broennimann, R. Buizza, P. Dalhgren, D. Dee, L. Haimberger, H. Hersbach, Y. Kosakam, M. Martin, P. Poli, N. Rayner, E. Rustemeier, D. Schepers, CERA-20C: A coupled reanalysis of the twentieth century. J. Adv. Model. Earth Sy. 10, 1172-1195 (2018).
84. Copernicus Climate Change Service (C3S), ERA5: Fifth generation of ECMWF atmospheric reanalyses of the global climate. Copernicus Climate Change Service Climate Data Store (CDS), accessed on 31 October 2019. https://cds.climate.copernicus.eu (2017).
85. H.J.B. Birks, Estimating the amount of compositional change in late-Quaternary pollen-stratigraphical data. Veg. Hist. Archaeobot. 16, 197-202 (2007)
86. D.H. Jewson, N.G. Granin, R.Y. Gnatovsky, S.F. Lowry, K. Teubner, Coexistence of two Cyclotella diatom species in the plankton of Lake Baikal, Freshwater Biol. 60, 2113-2126 (2015).
87. D.H. Jewson, N.G. Granin, A.A. Zhdanov, L.A. Gorbunova, N.A. Bondarenko, R.Yu. Gnatovsky, Resting stages and ecology of the planktonic diatom *Aulacoseira skvortzowii* in Lake Baikal, Limnol. Oceanogr. 53, 1125-1136 (2008).
88. A.W. Mackay, D.B. Ryves, D.W. Morley, D.H. Jewson, P. Rioual, Assessing the vulnerability of endemic diatom species in Lake Baikal to predicted future climate change: a multivariate approach. Global Change Biol. 12, 2297-2315 (2006).
89. A.W. Mackay, D.B. Ryves, R.W. Batterbee, R.J. Flower, D. Jewson, P. Rioual, M. Sturm, 1000 years of climate variability in central Asia: assessing the evidence using Lake Baikal (Russia) diatom assemblages and the application of a diatom-inferred model of snow cover on the lake. Global Planet. Change. 46, 281-297 (2005).
90. P. Šmilauer, J. Lepš, Multivariate analysis of ecological data using CANOCO 5, Cambridge University Press, Cambridge (2014).

91. D.H. Jewson, N.G. Granin, A.A. Zhdarnov, L.A. Gorbunova, R.Y. Gnatovsky, Vertical mixing, size change and resting stage formation of the planktonic diatom *Aulacoseira baicalensis*. Eur. J. Phycol. 45, 354-364 (2010).
92. S.N. Wood, Fast stable restricted maximum likelihood and marginal likelihood estimation of semiparametric generalized linear models. J. R. Stat. Soc. B 73, 3-36 (2011).
93. S.N. Wood, Generalized Additive Models: An Introduction with R, CRC Press (2017).
94. G.L. Simpson, Modelling Palaeoecological Time Series Using Generalised Additive Models. Front. Ecol. Evol. 6, 149 (2018).
95. G.L. Simpson. gratia: Graceful 'ggplot'-Based Graphics and Other Functions for GAMs Fitted Using 'mgcv'. R package version 0.4.1. https://CRAN.R-project.org/package=gratia (2020).
96. V.M. Muggeo, segmented: an R Package to Fit Regression Models with Broken-Line Relationships. R News, 8, 20-25. https://cran.r-project.org/doc/Rnews/ (2008).
97. G. Rzheplinsky, A. Sorokina, Atlas of wave and wind action in Lake Baikal. Gidrometeoizdat (1977) [in Russian].
